# Identifying causal gateways and mediators in complex spatio-temporal systems

**DOI:** 10.1038/ncomms9502

**Published:** 2015-10-07

**Authors:** Jakob Runge, Vladimir Petoukhov, Jonathan F. Donges, Jaroslav Hlinka, Nikola Jajcay, Martin Vejmelka, David Hartman, Norbert Marwan, Milan Paluš, Jürgen Kurths

**Affiliations:** 1 Potsdam Institute for Climate Impact Research, PO Box 60 12 03, Potsdam 14412, Germany; 2 Department of Physics, Humboldt University, Newtonstrasse 15, Berlin 12489, Germany; 3 Stockholm Resilience Centre, Stockholm University, Kräftriket 2B, Stockholm 11419, Sweden; 4 Department of Nonlinear Dynamics and Complex Systems, Institute of Computer Science, Academy of Sciences of the Czech Republic, Pod vodárenskou věží 2, Prague 8 18207, Czech Republic; 5 Department of Atmospheric Physics, Charles University, V Holešovičkách 2, Prague 8 18000, Czech Republic; 6 Computer Science Institute, Charles University, Malostranské náměstí, 2/25, Prague 1, 18000, Czech Republic; 7 Institute for Complex Systems and Mathematical Biology, University of Aberdeen, Aberdeen AB24 3UE, UK; 8 Department of Control Theory, Nizhny Novgorod State University, Gagarin Avenue 23, Nizhny Novgorod 606950, Russia; 9 Institute of Applied Physics of the Russian Academy of Sciences, Ul Ulyanova 46, 603950, Nizhny Novgorod, Russia

## Abstract

Identifying regions important for spreading and mediating perturbations is crucial to assess the susceptibilities of spatio-temporal complex systems such as the Earth's climate to volcanic eruptions, extreme events or geoengineering. Here a data-driven approach is introduced based on a dimension reduction, causal reconstruction, and novel network measures based on causal effect theory that go beyond standard complex network tools by distinguishing direct from indirect pathways. Applied to a data set of atmospheric dynamics, the method identifies several strongly uplifting regions acting as major gateways of perturbations spreading in the atmosphere. Additionally, the method provides a stricter statistical approach to pathways of atmospheric teleconnections, yielding insights into the Pacific–Indian Ocean interaction relevant for monsoonal dynamics. Also for neuroscience or power grids, the novel causal interaction perspective provides a complementary approach to simulations or experiments for understanding the functioning of complex spatio-temporal systems with potential applications in increasing their resilience to shocks or extreme events.

## Abstract (web summary)

Identifying regions important for spreading and mediating perturbations is crucial to assess the susceptibilities of complex systems such as the Earth's climate. Here the authors introduce a data-driven approach that identifies causal pathways, and apply it to a global atmospheric data set.

## Graphical abstract

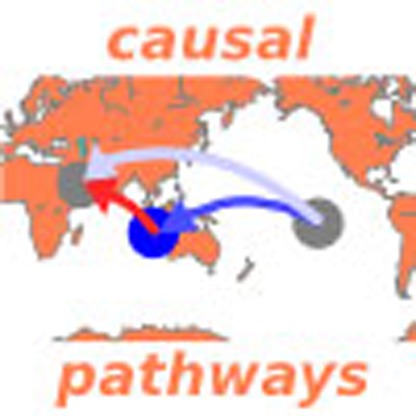

A complex system's susceptibility to perturbations may crucially depend on where such a perturbation enters the system and how it is propagated. In the climate system, perturbations such as volcanic eruptions, extreme events^1^,^2^ or anthropogenic manipulations such as air pollution and geoengineering^3^,^4^ may have very different global effects if the region they occur in is strongly connected globally. The huge volcanic eruption of Mt. Pinatubo in June 1991 had a large impact on global climate^5^ also because it is located in a climatologically sensitive region tied to atmospheric teleconnections, the tropical western Pacific^6^. Similarly, epileptic seizures in the brain^7^, blackouts in power grids^8^,^9^, epidemic spreading^10^,^11^ or the failure of certain banks in the financial system^12^,^13^ are key examples where subprocesses have a high cumulative effect on the whole complex system when perturbed, making them gateways of external influences spreading in the system. How can such subprocesses or regions be identified? Through which subprocesses are perturbations mainly mediated? These questions are key for understanding the dynamics and functioning of these systems, predicting their behaviour under perturbations and could help to make them more resilient. One way to address this problem is via active experiments, for example invasive brain stimulations in neuroscience (bearing ethical concerns) or by computer simulations, for example, in epidemic spreading models^11^ or tracer experiments in climate^14^. Such simulations are, however, only possible if the underlying physical equations are known and even then the corresponding calculations, for example in climate research, are computationally expensive and may not adequately represent important processes^15^. Here we follow the complementary approach of using the data alone to retrieve information about the interaction dynamics of the complex system (exploiting passive or natural experiments). Data-driven analysis within the framework of complex networks^16^ is a very active field of current research and has, among others, been applied to study the structure and function of complex systems in neuroscience^17^,^18^,^19^,^20^ and more recently also in climate research^21^,^22^,^23^,^24^,^25^,^26^.

To identify how important individual subprocesses are in spreading and mediating perturbations in such spatio-temporal complex systems with time series typically given on a spatial grid, the subprocesses or nodes first need to be reconstructed since the gridded time series are often not the variables of interest. Secondly, the analysis should be based on a network that more faithfully than pairwise statistical associations represents possible pathways of perturbation propagation, requiring a causal definition of network links able to distinguish direct from indirect interactions. Last, even if all links in a network were established to be ‘statistically causal', the toolbox of classical network measures is not rich enough for quantifying gateways and mediators of perturbations. Essentially, these measures—with many originating from the social sciences^27^—are based on a different definition of links, for example, two persons knowing each other, as opposed to dynamical interactions in a complex system. Hence, what is needed are quantitative measures that take into account the relative importance of causal pathways on which perturbations propagate in a complex system's interaction network.

Here, we present such an approach based on three steps: First, a dimension reduction of a gridded data set using the Varimax approach^28^,^29^ to a set of components representing relevant subprocesses defining the network's nodes. Secondly, a (multivariate) causal reconstruction of the network's links based on a causal discovery algorithm^30^,^31^,^32^ and, thirdly, a causal interaction quantification utilizing Pearl's causal effect theory^33^,^34^,^35^ to construct a causally weighted directed network on which we define network measures that are better suited for quantifying key regions of causal perturbation spread and mediation compared to classical network measures such as the node degree and betweenness centrality^36^. The extent to which such a data-driven analysis allows for a causal interpretation depends on the included variables, time resolution of the data and assumptions such as stationarity. We demonstrate the potentials of our method on a global data set of surface pressure as a representative characteristic of atmospheric variability. Applied to test specific hypotheses, we find that within this pressure system the climatic interaction mechanism between the East Pacific limb of the El Niño Southern Oscillation (ENSO)^37^,^38^, and the Arabian Sea region, relevant for the Indian Monsoon system, is mainly mediated via the Indonesian archipelago. This application of our method also incorporates more rigorously the concept of atmospheric teleconnections, which were previously defined based on pairwise correlations^39^,^40^. As an exploratory tool, the method identifies several strongly uplifting regions of major convergence of low-level air masses and high-level air uplifts above the tropical oceans. These subprocesses integrate incoming perturbations at the surface and transport them vertically into the higher troposphere from which they again influence other surface processes via atmospheric downdrafts. This mechanism explains the key importance of these regions as gateways of perturbation spread along causal pathways in the atmosphere. Our approach is of substantial value for several applications. In climate research it may allow to more efficiently allocate resources to understand, monitor and forecast these important subprocesses. For other applications, like epileptic seizure prevention, it may help to more reliably identify which brain regions are seizure foci to concentrate counter measures on. In summary, the novel causal interaction perspective provides a complementary first-step approach towards model simulations and experiments to better understand the dynamics and functioning of complex spatio-temporal systems and may help to inform design and engineering processes aiming at increasing their resilience.

## Results

### Dimension reduction and causal reconstruction

**Figure 1:**
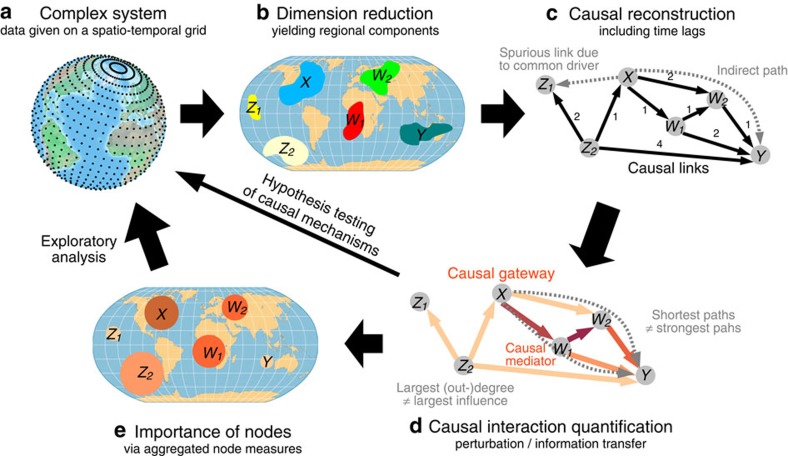
Schematic representation of our proposed approach illustrated for the complex system Earth. (**a**) Climatological variables such as sea-level pressure or surface temperature are typically provided as time series at locations on a regular grid. (**b**) In the first step of our approach, a Varimax-dimension reduction yields a small set of regional components (here denoted by *X*, *Y*, …) representing climatological subprocesses with corresponding time series. (**c**) In the second step of our approach, this smaller set of variables allows for a reconstruction of the causal network (black links, labels denote time lags). An important pitfall in non-causal networks (as constructed from pairwise association measures such as cross-correlation) is that links can be spurious due to a common driving by another process or due to transitivity effects leading to indirect paths (grey dashed arrows in **c**). Perturbations cannot propagate along common-driver links (for example, between *X* and *Z*_1_) affecting network measures like the degree. Further, indirect paths such as from *X* to *Y* affect shortest path lengths in non-causal networks. (**d**) In the third step of our approach, the aim is to directly quantify the causal effect between pairs of components based on the causal network (Tigramite approach) and detect through which components and how much the causal effect is mediated. In the linear framework studied here this can be achieved by causal effect measures based on suitable link weights, where the weight of a link, for example *X*→*W*_1_, indicates the causal effect of a 1 s.d. perturbation in *X* on *W*_1_ (see Fig. 3b for a formula relating link weights to causal effects). Binary causal networks do not properly account for different link strengths which affects classical network measures (grey highlights in **d**). This analysis can be used to test specific hypotheses, but also to construct aggregate node measures (**e**) to identify components with high cumulative causal effect either as sources (causal gateways) or as intermediate nodes on causal pathways (causal mediators).

In the following, we explain and illustrate each of the three steps (see Fig. 1) with a climate example. More technical details are given in Methods.

**Figure 2:**
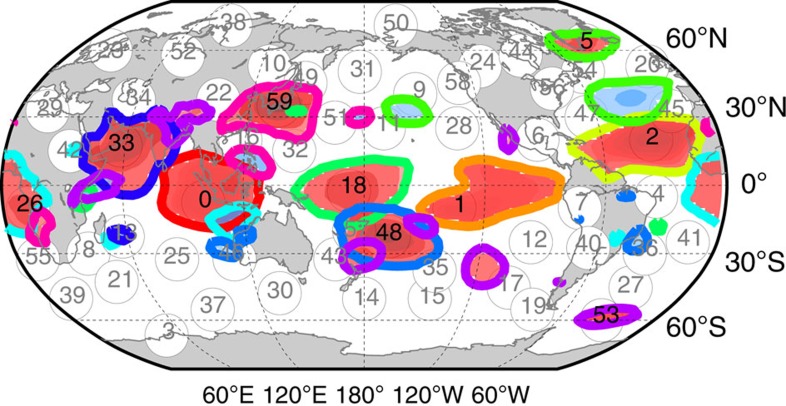
Dimension reduction of an atmospheric data set. Map of the 60 Varimax components, enumerated from 0 to 59 (grey nodes). The location of the nodes is determined by the largest absolute spatial loading. For selected components (all components shown in Supplementary Figs 1, 2), we show the core 98% area of loadings. The colour of the surrounding line identifies the parts belonging to one component, sometimes several regions belong to one component (examples include dipole patterns). Some components can be associated with well-known climatic processes like the El Niño Southern Oscillation^37^ (ENSO, No. 0 representing the western uplift and No. 1 the eastern downdraft limbs), or the North Atlantic Oscillation (NAO, No. 5 with a pronounced dipole structure over the Icelandic Low and the Azores High). Others are related to global monsoon systems: No. 33 in the Arabian Sea high-surface-pressure sector of the Indian Monsoon region^53^ and No. 26 in the tropical Atlantic West African Monsoon system^43^. Not all components are regionally confined, for example, No. 53 with loadings in the South Atlantic as well as in the Himalayas.

In climate research, spatio-temporal data sets are typically given on a regular grid. Here we consider a reanalysis data set of surface pressures^41^ for the period 1948–2012. At a resolution of 2.5° in latitude and longitude, the data set consists of 10,512 grid points with 3,339 samples for each time series on a weekly timescale. But towards an interpretation of perturbation propagation or information transfer^42^, such individual grid points are not the entity of interest, because they do not represent distinct climatological processes. For example, processes like ENSO require a special decomposition of the data fields for an efficient description of their spatio-temporal structure^43^. Also from a statistical perspective, a large number of variables with comparatively few observations presents an estimation problem^44^. The first step of our approach, therefore, is aimed at obtaining a small set of components that represent relevant subprocesses of the complex system. We choose Varimax-rotated principal components^28^,^45^ here, combined with a subsequent significance test^29^,^46^ to exclude components merely representing noise. For the atmospheric pressure data set, this dimension reduction algorithm yields a set of 60 components (cf. Methods and ref. 29). As shown for selected components in Fig. 2 (all components shown in Supplementary Figs 1, 2), the corresponding regions well represent several important climatological subprocesses. As further discussed in Methods, all components are anomalized (seasonal cycle removed from the mean and variance) and standardized. Here we study intraseasonal interactions at a weekly time resolution.

**Figure 3:**
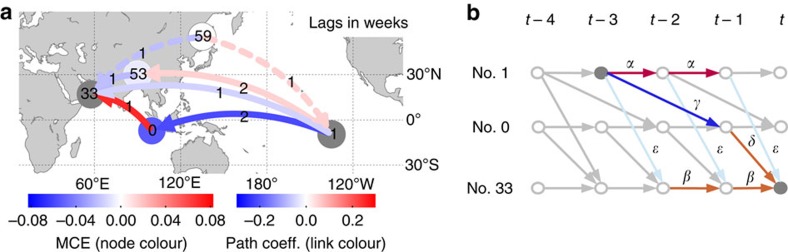
Results for the teleconnection between ENSO and the Indian Ocean within the causal network of pressure components. (**a**) Exemplary selection of causal paths (solid lines) for the effect of No. 1, the eastern limb of ENSO, on No. 33 in the Arabian Sea at a lag of 3 weeks via components No. 0 above the Indonesian archipelago and No. 53 over East Asia. The edge colour corresponds to the path coefficient (labels denote the lags in weeks) and the node colour to the MCE. The causal measures exclude confounding effects due to common drivers of both processes, such as No. 59 (dashed links), but also from component No. 1 at lags further in the past as the more detailed time series graph representation reveals. (**b**) Time series graph depicting only links belonging to the most relevant causal paths (line color indicating the path coefficient) between Nos. 1 and 33 (grey nodes) at lag *τ*=3 weeks via No. 0. As further discussed in Methods, one can estimate the total causal effect by summing over the products of path coefficients (link labels) along each path: 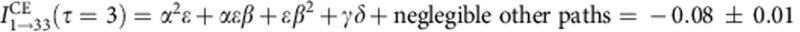. The mediated effect via component No. 0 is given by the only path through this node: 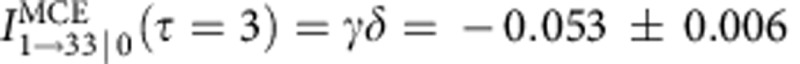. The s.e. are computed from a residual bootstrap (see Methods). Intermediate components can also counteract the total causal effect if MCE is of different sign than CE.

Given that an external perturbation occurs in one of these components representing a certain subprocess: On which paths can it propagate and which other subprocesses can it possibly reach? Suppose a perturbation enters in subprocess *X* in the example in Fig. 1c, then it can only reach nodes further ‘downstream' on causal paths like *W*_1_, *W*_2_ and *Y*, but not *Z*_1_, even though they are statistically associated. Such spurious links can be unveiled by causal discovery algorithms^30^,^47^ which iteratively test whether an association can be explained by another process in the network. Note that this notion of causality is only to be interpreted with respect to the included variables and unobserved drivers can still cause spurious links. The causal reconstruction steps are detailed in Methods (see also Supplementary Fig. 3). In essence, for the second step of our approach we employ a causal discovery algorithm adapted to time series^31^,^32^ and a subsequent thresholding step to study the robustness of all further analyses at different link densities. This approach yields the causal time series graph^31^,^32^,^48^ which is a special type of a graphical model^49^ and encodes the conditional dependencies of the components at different time lags. For the example of the ENSO—Indian Ocean teleconnection studied next, Fig. 3 depicts two different representations: Fig. 3b shows the time series graph on which causal paths and the measures of causal effect (the third step of our approach) are based, while the aggregated causal network shown in Fig. 3a can be better visualized.

In our climate application, we consider time lags up to *τ*_max_=4 weeks, since we are interested in atmospheric interactions where dependencies typically decay within a month^50^, but our results are robust also for longer time lags. Contemporaneous associations (possibly because of unobserved common drivers or faster interactions) can be represented as undirected links in the time series graph^31^,^32^, but these are not taken into account here since they are not regarded as causal. In the following, we discuss results for a link density of 20% in the causal network consisting of *N*=60 components, but our main results are also robust for link densities between 10 and 50% and other parameters of the method.

### Quantifying causal effect

The causal time series graph allows to qualitatively determine which causal paths a perturbation can possibly take. Now we employ measures to quantify the causal effect of hypothetical perturbations and their mediation along causal paths, exemplified on the teleconnection mechanism by which component No. 1 in the East Pacific ENSO region influences component No. 33 describing surface pressures in the Arabian Sea with relevance also for the Indian Monsoon rainfall^37^,^38^,^43^ (see component regions in Fig. 2).

We approach this problem by using measures of causal effect in the framework of structural equation modelling^33^,^35^. With the reconstructed time series graph as a causal hypothesis, we fit a linear regression model to the multivariate component time series **X** with non-zero coefficients for every link in the time series graph. The standardized regression coefficient for a direct causal link 
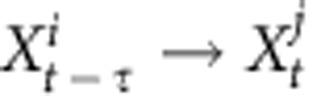
 between two components 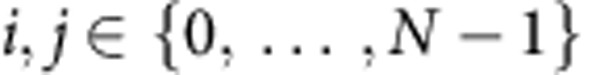 at lag *τ* (in weeks) is then called the path coefficient^33^,^51^. This makes the time series graph a causally weighted directed network. The matrices of path coefficients between all components are shown in Supplementary Fig. 4.

Rather than studying only causal effects between adjacent nodes in the causal time series graph, here we are interested in the total causal effect (CE) also along indirect causal paths. Under certain assumptions (see Methods), the CE between two components *i* and *j* at lag *τ*, denoted 
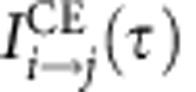
, can be evaluated by summing over the products of the path coefficients along each causal path^35^ and carries the causal interpretation as the expected change in *X*^*j*^ (in units of its s.d. and relative to the unperturbed regime) at time *t* if *X*^*i*^ was perturbed at time *t*–τ by a one s.d. delta peak. The matrices of CEs between all components are shown in Supplementary Fig. 5. Similarly, the mediated causal effect (MCE) 
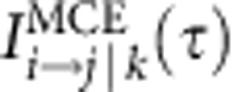
 via another component *k* can be measured by summing only over those paths that pass through component *k* (at any lag). These measures are now illustrated for the teleconnection mechanism between components Nos. 1 and 33.

In the atmospheric pressure system we find 31 indirect causal paths between the Eastern ENSO component No. 1 and the Arabian Sea component No. 33 at a lag of 3 weeks (only a selection via components No. 0 above the Indonesian archipelago and No. 53 over East Asia shown in Fig. 3a). The total CE sums up to −0.08±0.01 here, implying that a perturbation of 1 s.d. in the East Pacific yields a decrease in No. 33 of ∼8% in units of its s.d. (note that component No. 1 has deviations of several s.d. from the seasonal mean). Further, we find that component No. 0 above the Indonesian archipelago mediates −0.053±0.006 of that effect, here resulting from the causal chain No. 1 
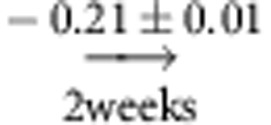
 No. 0 
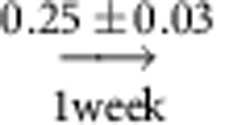
 No. 33. This MCE explains more than 60% of the total CE while other paths, for example via East Asia (No. 53), contribute less than 10%. For comparison, counting just the fraction of causal paths passing through a given node, in analogy to betweenness centrality^36^, here yields 1 from a total of 31 paths for No. 0 and, for example, 8 for component No. 35, even though the latter's mediated effect is much weaker. We estimated the CE for a link density of 20% here, in Supplementary Table 1 we show that our results are largely robust to this choice. On the other hand, we find that the interaction was much weaker in the first half of the data set (1948–1980).

Climatologically, our present analysis implies that of the many possible climatic mechanisms linking sea-level pressure anomalies in the ENSO region to pressure variability west of India, only the mechanism via No. 0 is relevant, at least within the intraseasonal timescale of the atmospheric surface pressure system and integrated over all seasons. Note that conclusions about an effect of ENSO on the Indian Monsoon are also complicated by the apparent non-stationarity of this relationship^52^. More detailed analyses taking into account additional climatological variables such as temperature, only certain seasons (for example, during El Niño phases), and filtering out non-relevant time scales (such as from oceanic drivers) can provide more accurate estimates of CEs for more specific climatological hypotheses.

### Causal gateways and mediators

The foregoing case study introduced the CE measures and is an example of causal modelling for testing specific hypotheses about interaction mechanisms. Now we study aggregated node measures based on the causally weighted directed network in a more exploratory analysis to identify the importance of components as gateways for spreading and mediating hypothetical perturbations in the network.

**Figure 4:**
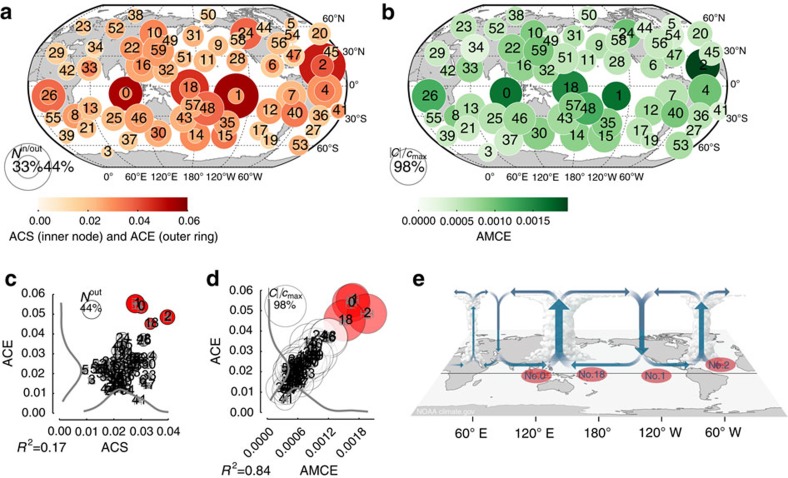
Aggregated measures of causal effect in the causal network of pressure components. (**a**) For each node/component, the fraction of components 
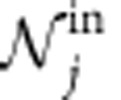
 affecting component *j* with 
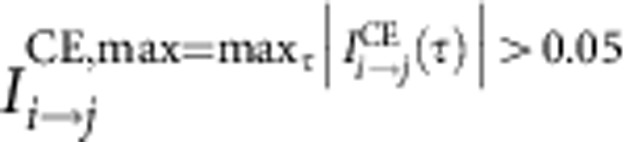
 (that is, a 5% effect taken at the lag with maximum absolute effect) scales with the inner node diameter and the fraction of processes 
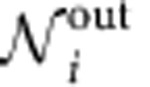
 reached by component *i* scales with the width of the outer ring. The colours denote the ACS 
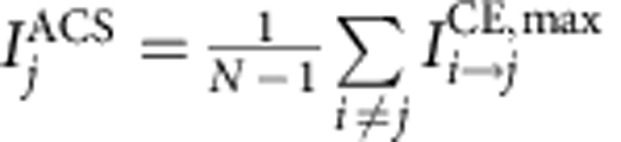
 (inner node) and average causal effect 
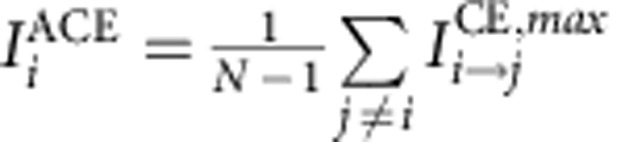
 (outer ring). (**b**) For each node/component *k*, the fraction of pairs 
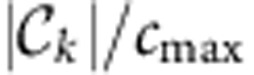
 (with *c*_max_=(*N*–1)(*N*–2)) where this node is a mediator on a causal path (up to a lag of τ_max_=4 weeks) scales with the node diameter. The colour shows the average mediated causal effect 
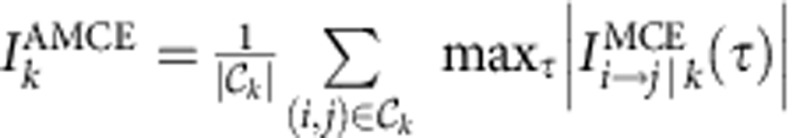
. (**c**) and (**d**) depict ACE versus ACS and AMCE, respectively. The thick grey lines denote the density of the marginal distributions (arbitrary units). The error bars show the standard errors computed from a residual bootstrap (see Methods). (**e**) shows a schematic view of the global equatorial Walker circulation during normal conditions. Note that during La Niña events the uplift and downdraft regions are enhanced and during El Niño events they are reversed. Our analysis constitutes an average over all seasons (adapted with permission from NOAA Climate.gov drawing by Fiona Martin). The red components in (**c**,**d**) with high ACE, ACS and AMCE correspond to major convergence regions that integrate incoming perturbations at the surface and transport them vertically into the higher troposphere from which they influence other components via atmospheric downdrafts. In Supplementary Fig. 6 we depict more detailed results for these major gateways.

As aggregated first-order measures of CE, we consider the matrix of CEs between all pairs of components (Supplementary Fig. 5) taken at the lag with maximum absolute effect 
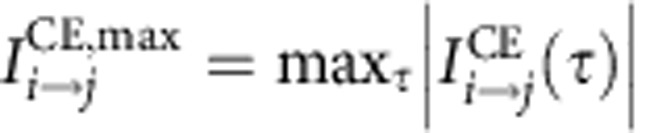
. Then we define the mean along each column as the average causal effect (ACE) that a component has on the rest of the system and the row-mean as the average causal susceptibility (ACS) as a measure of how sensitive a component is to perturbations entering in other parts of the system. To measure how strong a subprocess mediates CEs spreading throughout the complex system, we propose the average mediated causal effect (AMCE) of a component *k* by averaging the previously defined MCE over all interaction pairs with causal paths through *k* (more details in Fig. 4 and Methods). As opposed to a path-based network measure like betweenness centrality^36^, AMCE depends not so much on the number of paths through a given component, but more on how strong the CE along these paths is.

In Fig. 4 we depict ACE, ACS and AMCE for the atmospheric pressure system. Although the distribution of susceptibilities is quite broad (Fig. 4c), few components have a very strong ACE (red nodes Nos. 0, 1, 2, 18) and are also rather susceptible. These components reach a large fraction of processes (node size in Fig. 4a,c) and correspond to processes in the tropical oceans (No. 0 over Indonesia and the East Indian Ocean, No. 2 in the Atlantic, Nos. 1 and 18 in the East and West Pacific). A one s.d. perturbation entering these processes has a large effect of up to 0.3 on other processes and each of them drives more than 10 other processes with a CE of at least 0.1 (Supplementary Fig. 6). These components, thus, act as major gateways of perturbation spread and also belong to the most susceptible processes being causally driven by ∼20–30% of the other components with an ACS above 0.05.

Figures 4b,d demonstrate that there is not much correlation between the fraction of interactions with a path through a certain component (node size) and its AMCE (*R*^2^=0.36). Components Nos. 0, 1, 2, 18 (but also Nos. 26 and 48) are the most dominant causal mediators being involved in more than 80% of all interactions with an AMCE between 0.0015 and 0.002. Note that the average non-zero CE between any pair is only about 0.02. In Supplementary Figs. 7, 8 we show that these results are robust for different link densities and other parameters of the method, in particular the maximum lag *τ*_max_ and the significance level in the algorithm. The results are also robust if only the first (1948–1980) or second half (1981–2012) of the data set is used.

Climatologically, components Nos. 0, 1, 2, 18 correspond to major convergence regions with ascending motion of air masses (cf. Fig. 4e)^43^,^53^. In particular, component No. 0 (and to a smaller degree No. 18) represents the uplifting western limb of the Walker circulation over Indonesia. This region is one of the strongest atmospheric convergence zones where moist air masses rise up and affect global tropical and extratropical climate via teleconnections in the upper troposphere. Component No. 2, located in the tropical Atlantic, also features strong uplifting deep convection^43^,^53^. The core ENSO region represented by component No. 1 plays a double role depending on the state of the ENSO system^37^,^38^. During normal ENSO conditions (depicted in Fig. 4e) it is a region of descending upper tropospheric air masses and, thus, not as much governed by surface pressures. During El Niño events, on the other hand, it is a region of strong uplifts. These effects are mixed in our analysis and more detailed studies can further distinguish seasons to obtain a more precise picture of seasonal climatic interactions. In summary, these strongly uplifting regions integrate incoming perturbations at the surface and transport them vertically into the higher troposphere from which they again influence other surface processes via atmospheric downdrafts. This mechanism explains the key importance of these regions as causal gateways and mediators of perturbations spreading in global climate via atmospheric teleconnections. Our analysis considered delta peak perturbations of 1 s.d. Since often perturbations reach extreme deviations of several s.d.^1^,^2^,^54^, and, even more importantly, multiple perturbations can accumulate, these findings reflect the large global influence of these regions.

## Discussion

We have introduced a three-step approach for the analysis of multivariate spatio-temporal data sets, consisting of a dimension reduction, causal reconstruction, and CE quantification to identify subprocesses in complex systems that are important gateways for spreading and mediating perturbations entering the system in one subprocess. While this approach lends itself also to other spatio-temporal complex systems such as the brain^18^, for applications to financial data or food webs, the causal reconstruction can already be applied to, for example, economic indices or species abundances, and in complex systems like power grids or transportation networks, the first two steps of our approach could be skipped since the network structure is naturally given.

The causal quantification approach takes classical analyses of functional brain networks^18^ or climate networks^21^,^23^, which were previously mostly based on pairwise association measures, to a new level. Consequently, our proposed node measures can be seen as dynamical and causal alternatives to classical measures for functional networks such as the degree or betweenness centrality. In Supplementary Fig. 9 and Supplementary Note 1 we compare our results with these measures where we find that the latter have only weak predictive power (*R*^2^≈0.4) for perturbation propagation and mediation. The pathway analysis goes substantially beyond pure causal network reconstructions^24^,^32^ and also provides, for the first time, a stricter statistical approach for characterizing atmospheric teleconnections, which are of paramount importance for studies of climate change and in particular climate extremes^55^, and which were previously formulated more phenomenologically based on (lagged) correlations^39^,^40^,^56^.

Like any data-driven approach our method is limited by several assumptions: causal sufficiency^30^,^33^ assumes that the common drivers of all variables are taken into account and the causal Markov condition assumes all ‘error terms' of the nodes in the time series graph to be independent. In our climate analysis, for example, we only excluded common drivers from within the pressure system, but on larger monthly time scales the underlying sea surface temperature field certainly interacts with the faster atmospheric pressure field over the oceans^57^ and can confound the assessment of CEs. It is, therefore, important to interpret CEs only relative to the variables that were taken into account. A further complication are CEs that are faster than the weekly resolution considered here, which appear as contemporaneous in our analysis, but are not taken into account since they are not regarded as causal links. Our weekly time resolution reflects a balance between resolving causal directionality and a multiple testing problem if too many lags are considered (in our example 30 days). Also the interplay of different time scales^58^,^59^ could be further addressed, for example by singular spectrum analysis^45^, and one could possibly also account for time-varying time delays of interactions^60^. To estimate the time series graph and CEs from the observed time series, we assume stationarity such that these properties do not change over time. More detailed research questions can take into account non-stationarity, for example, due to seasonality in climate (here we used the whole time series). While the linear approach can also be adapted from delta-peak perturbations to more general scenarios with multiple or different types of perturbations^35^, the perturbations must be small enough to conserve the dynamics and causal structure of the system such that the conditional distributions remain the same^35^. The effects of large unprecedented perturbations cannot be predicted from observed data alone. We introduced the method using simple linear measures here, but the framework can to some extent also be implemented with nonlinear quantifiers, for example using information-theoretic measures^42^,^61^.

We see the proposed method as a complementary first-step approach towards model simulations and experiments to help guide decision making in several ways: in climate, the knowledge of subprocesses or regions with large perturbative effect, either as gateways or mediators, can help to optimally design computationally expensive simulations such as tracer experiments^14^, geoengineering impact assessments^3^,^4^, or extreme event attribution studies^62^. Such experiments allow to conduct counterfactual analyses, for example, with and without anthropogenic influences, to conclude on necessary and sufficient CEs^63^. In neuroscience, it could help to optimize therapeutic interventions for preventing seizures by targeting selected brain regions with large CE or mediating CE. In power grids, nodes with strong mediating effect are the ones that one would best block to prevent a blackout perturbation from spreading throughout the network. Summarizing, the novel causal interaction perspective provides a general approach to better understand the possible influence of perturbations on complex spatio-temporal systems and may guide further research to inform design and engineering processes aiming at increasing their resilience against shocks or extreme events.

## Methods

### Data and software availability

The climatological reanalysis data set^41^ studied here can be downloaded from http://www.esrl.noaa.gov/psd/data/gridded/data.ncep.reanalysis.html. Code for the dimension reduction step is available from co-author M. Vejmelka at https://github.com/vejmelkam/ndw-climate/blob/master/scripts. A Python software script by J. Runge to estimate the causal network can be obtained from http://tocsy.pik-potsdam.de/tigramite.php.

### Dimension reduction

Our dimension reduction approach is based on Varimax-rotated principal components^28^,^45^ and a subsequent significance test to eliminate components merely representing noise^29^. As further discussed in ref. 29, the rotation of principal components maximizes the sum of the variances of the squared principal component weights (loadings) and better represents regionally confined processes than principal components. The data preprocessing steps to obtain the component weights are discussed in more detail in ref. 29, here we give a brief summary: Monthly gridded time series are first anomalized to remove the annual cycle not only from the seasonal means but also from the seasonal variance. After a linear detrending, the covariance matrix is estimated on cosine-transformed data to account for the area a grid point represents (poles are excluded), and the eigenvectors are computed. These are then rotated using the Varimax criterion^28^ and a limited number of components is selected based on a comparison of eigenvalues of original data (not components) to those from surrogate data which preserve the autocorrelation structure, but destroy dependencies between the grid point time series. Here this algorithm yields *N*=60 significant components. Finally, the component weight matrix is multiplied with the daily original gridded time series (that have been preprocessed by anomalization in mean and variance, standardization, linear detrending and cosine transform as above), and the daily component time series are aggregated to a weekly resolution which reflects a balance between causal time resolution and the multiple testing problem in the causal reconstruction step. In contrast to principal components, where the diagonal entries corresponding to the eigenvalues can be interpreted as the explained variability, for rotated principal components, the off-diagonal entries are not zero anymore and one cannot simply attribute an ‘explained variance' to each component. We enumerate the components by the entry on the diagonal starting with the largest value (component No. 0). Here monthly time series were used for the extraction of the components for computational reasons. Carrying out the decomposition directly on the daily or weekly time series might have provided a slightly different set of components, as the decomposition would also take into account higher frequency variability. In Supplementary Figs 1, 2 we show the loadings and time series of all components.

### Causal reconstruction

To reconstruct the causal network from the component time series, we utilize a causal discovery algorithm^31^,^32^ which is based on the PC algorithm (named after its inventors Peter Spirtes and Clark Glymour^30^). This algorithm can be used in an information-theoretic framework^31^ as well as employing linear partial correlation^24^,^32^. Here we choose the linear approach as a first-order approximation. The significance level used in the causal algorithm is not a very reliable indicator for the final significance level of causal links because links are tested sequentially and, therefore, Bonferroni corrections cannot be easily applied. To overcome this problem, we use the causal algorithm only as a variable selection for a subsequent ‘causal regression'. The time series graph reconstruction, thus, consists of three steps as described below.

First, variable selection of the causal parents: The parents 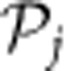 of each component *j* are selected with the causal algorithm described in ref. 32 and Supplementary Note 2. The algorithm's parameters here are: maximum time lag *τ*_max_=4 weeks, (two-tailed) significance level *α*=0.001 (Student's *t*-test), initial number of conditions *n*_0_=3. For the causal algorithm to consistently converge to the true parents, one needs to assume causal sufficiency and the causal Markov condition, that is, the independence of error terms driving each subprocess, and faithfulness^30^ which guarantees that the graph entails all conditional independence relations true for the underlying process and can be violated in certain pathological cases^30^. Since we estimate partial correlations from time series data here, we also assume stationarity. Ref. 64^64^ discusses the computational complexity of the algorithm. In Supplementary Fig. 3b we show the distribution of the number of parents for every component (black dashed line) and in Supplementary Tables 2, 3 we list the parents for all components (median number of parents is 8).

Secondly, estimating the causal regression matrix: the lagged causal regression matrix **C**(*τ*) of shape (*N*, *N*, *τ*_max_) is estimated using the above selected parents by


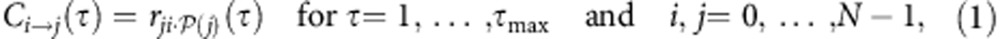


where *r* denotes the standardized regression coefficient of component 
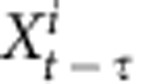
 in the multiple regression model of 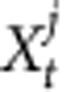 on 
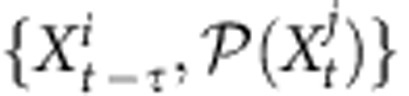
 using ordinary least squares regression. For infinite sample sizes, these ‘causal' regression coefficients would be non-zero only for the parents of each component as estimated with the algorithm. In Supplementary Fig. 3a we show the sorted coefficients for every component *j*. The largest coefficients are typically associated with the past lag of a component. After a sharp decay, most of the coefficients have absolute values below 0.1

Thirdly, constructing the time series graph: The causal time series graph is constructed from thresholding the causal regression matrix with cross-links (*i*≠*j*) and autolinks (*i*=*j*) defined by


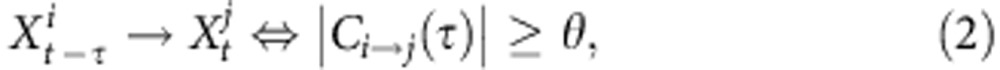


with the threshold *θ* chosen to obtain a given link density in the corresponding aggregated causal network (Supplementary Fig. 9b), that is, 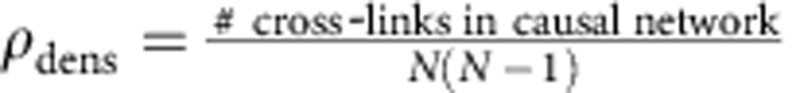, where autolinks are not counted and multiple links between two components are only counted once. For the link density *ρ*_dens_=0.2 analysed here, there are 708 such links, while the time series graph, thresholded at *θ*=0.0585, has a link density of 0.062. Because of the assumed stationarity, the subscript *t* in equation (2) can be dropped. For the network analysed in the main article at 20% link density, the median of parents in the time series graph is 14 which determines the non-zero coefficients in the CE-estimation model (4) described below. Supplementary Fig. 3b shows the distribution of parents for different link densities.

### Causal effect estimation

There are different ways to use the reconstructed causal network to further quantify causal interactions between subprocesses. We call the general idea to use the time series graph for quantifying general causal interactions the Tigramite approach (time series graph-based measures of information transfer), which is also the abbreviation of the accompanying software package. Here we consider a measure *I* to quantify the linear CE of perturbations for its reliable estimation and interpretability, generalizations in an information-theoretic framework are discussed in refs 42, 61.

The approach is based on the CE estimator for multivariate time series proposed in ref. 35 in a linear application of Pearl's causal framework^33^, considering delta-peak perturbations (called atomic interventions in ref. 35). Within this framework, the CE of a perturbation of setting 
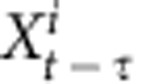
 to x* on 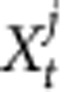 is given by


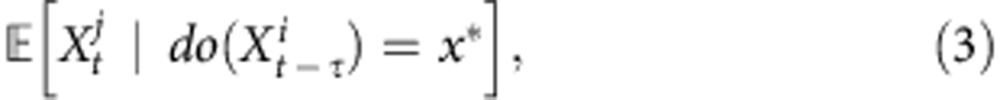


where 
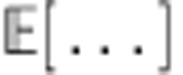
 denotes the expectation. It is important to note that the *do*-operator does not pertain to a conditional expectation, but refers to the experiment of intervening in the system and forcing the variable to a certain value. From observational data, this effect can only be estimated (or identified) under certain assumptions^33^,^35^. Here we assume a linear model based on the reconstructed time series graph with all relevant variables (or confounders) included (thus, satisfying the back-door criterion^33^,^35^):


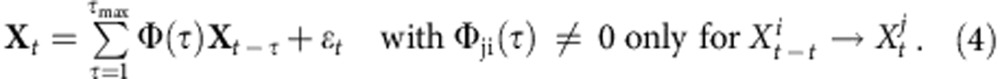


Note that we do not fit a full autoregressive model here, but a sparse one where we estimate only those coefficients corresponding to causal links in the time series graph (including cross- and autolinks). A standardized coefficient **Φ**_*ji*_(τ) is called path coefficient^34^,^51^ and stands for the change in the expectation of 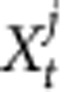 (in units of its s.d.) induced by raising 
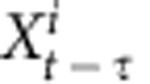
 by 1 s.d., while keeping all other parents of 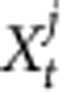 constant. The matrices of path coefficients between all components are shown in Supplementary Fig. 4. Then the CE of a perturbation x*=1, that is, a 1 s.d. for the standardized component time series, is given by^35^


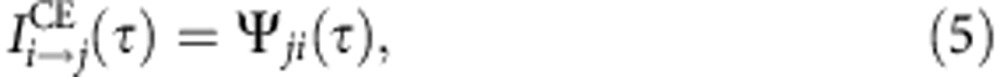


where **Ψ**(*τ*) can be iteratively computed from matrix products of the estimated coefficient matrices **Φ**(*τ*) by


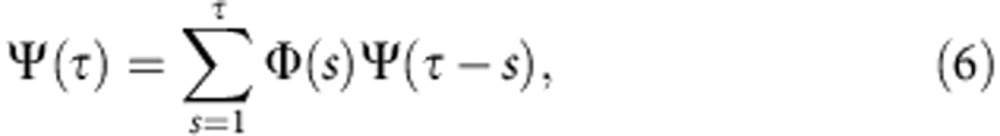


for example,


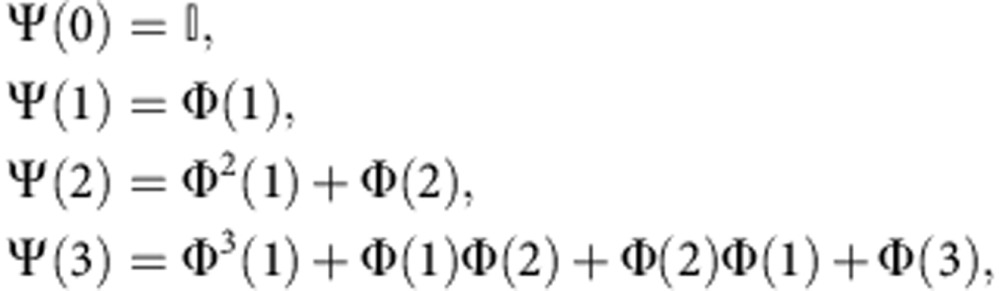


where 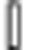 is the identity matrix. An entry **Ψ**_*ji*_(τ) here yields the sum over the products of path coefficients along all causal paths as explained for the climate example in Fig. 3. The framework also allows to encompass more complex types of perturbations such as the effect of multiple perturbations^35^. In Supplementary Fig. 5 we depict the matrices of CEs between all pairs of components for all considered link densities.

The MCE through a component *k* is defined as the sum over the products of path coefficients only along causal paths through *k*. From the matrices **Ψ** it can be derived as


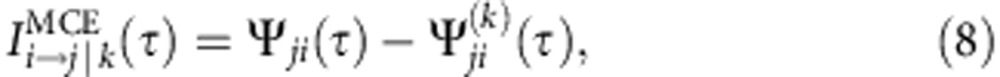


where **Ψ**^(*k*)^(*τ*) is computed from equation (6) with modified path coefficient matrices **Φ**^(*k*)^(*τ*) where all links towards component *k* are set to zero,


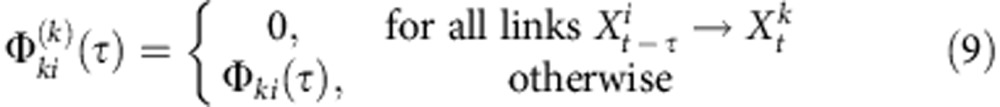


which blocks all paths through component *k* at any lag. The causal interpretation is that an indirect effect via the component *X*^*k*^ measures the change we would see in 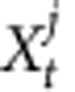 while holding 
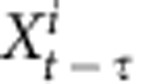
 constant and setting component *X*^*k*^ to whatever value it would have obtained under a unit change in 
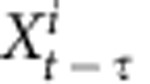
33,65.

### Aggregated measures

For the aggregated causal effect measures ACE and ACS we are interested not so much in the lag at which the interaction occurs and, therefore, base these measures on the lag with maximum effect:


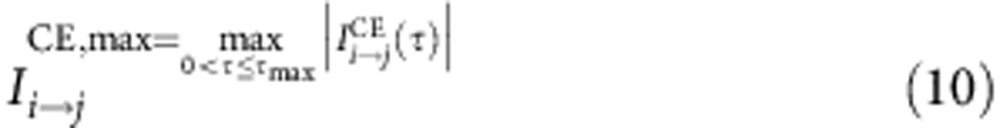



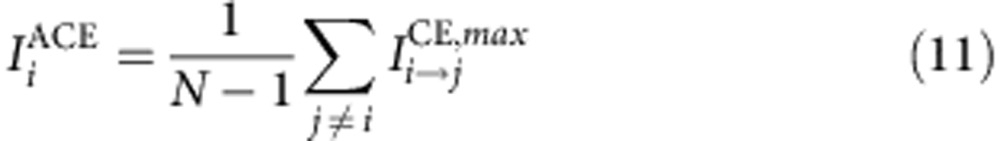



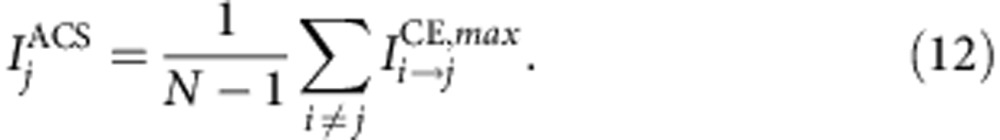



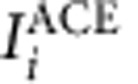
 quantifies by how much (in units of its s.d.) any of the *N*–1 remaining components is changed on average by a one unit increase in component *i* (at the lag with maximum absolute effect). This serves as a quantitative measure of how much a component is a gateway of perturbations. 
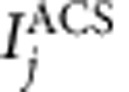
, on the other hand, measures by how much a component *j* is changed on average by a one unit perturbation in any of the *N*–1 remaining components. Further, we denote the fraction of components that *i* is influencing with 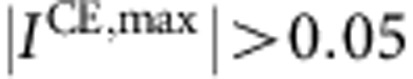 by 
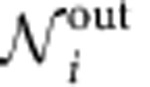
 and as 
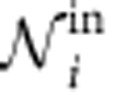
 the fraction of components that *j* is affected by, at any lag within 0<τ≤τ_max_. Normalizing ACE and ACS by these quantities results in a measure that is not as robust as desired because it depends on the chosen threshold. In Supplementary Fig. 7 we show ACE versus ACS for different network link densities and reconstruction parameters (maximum lag *τ*_max_, significance level α), and also for different segments of the data set.

The AMCE is based on causal paths passing through a given node:


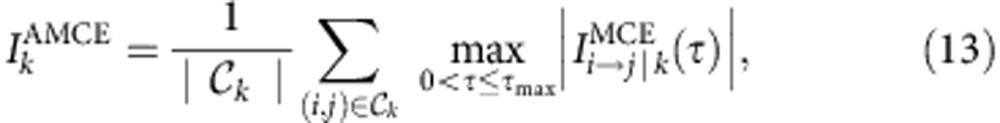


where 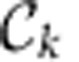 is the set of interactions between all non-identical pairs *i*, *j*≠*k* at all lags 0<τ≤τ_max_ where *k* is an intermediate component (at any lags) and 
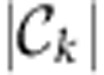
 denotes its cardinality. Here we take the absolute value 
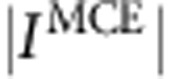
, but one could further distinguish between enhancing (where the sign of MCE equals that of CE) and counteracting (opposite signs) effects. In general, there can be maximally *c*_max_=(*N*–1)(*N*–2)=3,422 interacting non-identical pairs and in Fig. 4b,d we depict the fraction of interaction pairs 
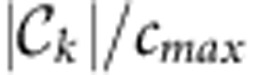
 where a component *k* is an intermediate node as the size of the nodes. In Supplementary Fig. 8 we show ACE versus AMCE as in Fig. 4d for different setups.

### Uncertainty quantification

To estimate the standard errors for all causal effect measures considered above, we employ a residual-based bootstrap procedure^66^. Each bootstrap surrogate 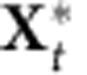 is constructed from running model (4) with a joint random sample 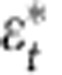 (with replacement) of the original multivariate residual time series 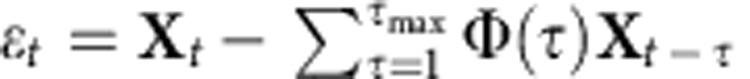 and with the original coefficient matrices **Φ**(τ),


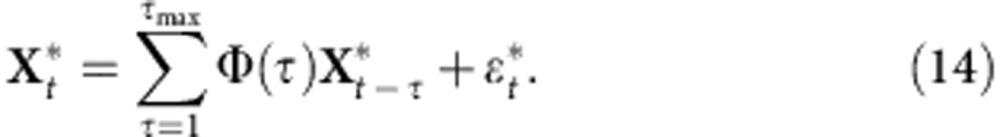


From this bootstrap surrogate time series, Φ*(τ) is estimated from which the other quantities are derived. We use 200 bootstrap surrogates here to estimate the standard errors of all quantities defined above (given as ± in the main text as well as Supplementary Table 1 and as error bars in scatter plots in Fig. 4 and in the Supplementary Figs 7, 8).

## Additional information

**How to cite this article:** Runge, J. *et al*. Identifying causal gateways and mediators in complex spatio-temporal systems. *Nat. Commun.* 6:8502 doi: 10.1038/ncomms9502 (2015).

## Supplementary Material

Supplementary Information Supplementary Figures 1-9, Supplementary Tables 1-3, Supplementary Notes 1-2 and Supplementary References
