# Supplementary material for: Identifying causal gateways and mediators in complex spatio-temporal systems
Source: Nat Commun. 2015 Oct 7;6:8502. doi: 10.1038/ncomms9502 (PMC4633716; doi:10.1038/ncomms9502)
Supplement: Supplementary Information — Supplementary Figures 1-9, Supplementary Tables 1-3, Supplementary Notes 1-2 and Supplementary References [file ncomms9502-s1.pdf]

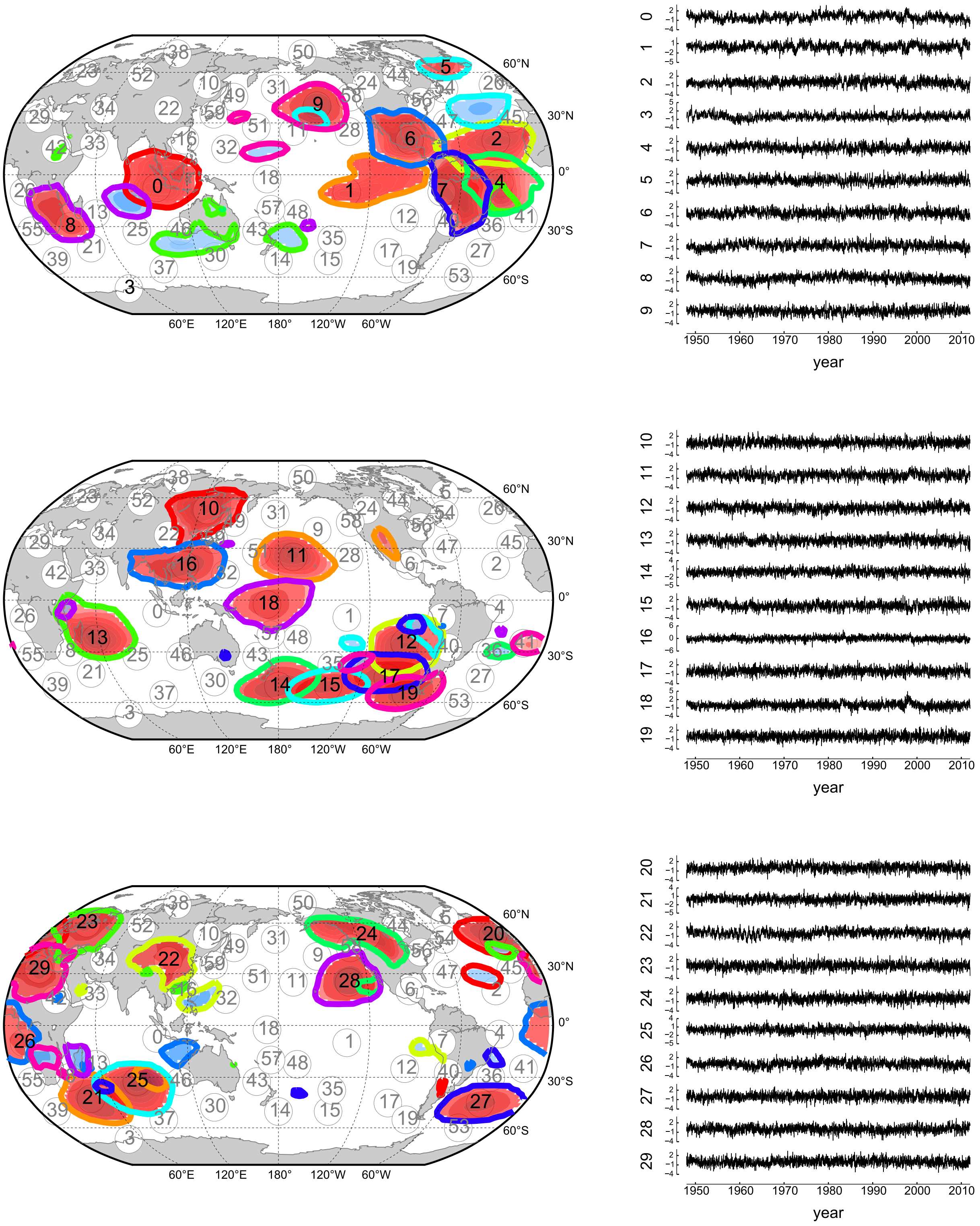

Supplementary Figure 1: Loadings and standardized time series of all components enumerated from 0 to 59. The location of the nodes is determined by the largest absolute weight (or spatial loading) and only the core (98% of the area-adjusted weight distribution) region of loadings is plotted. The color of the surrounding line identifies the parts belonging to one component. Note that the seasonal cycle has been removed from the mean as well as the variance. Some components feature extreme values of several standard deviations.

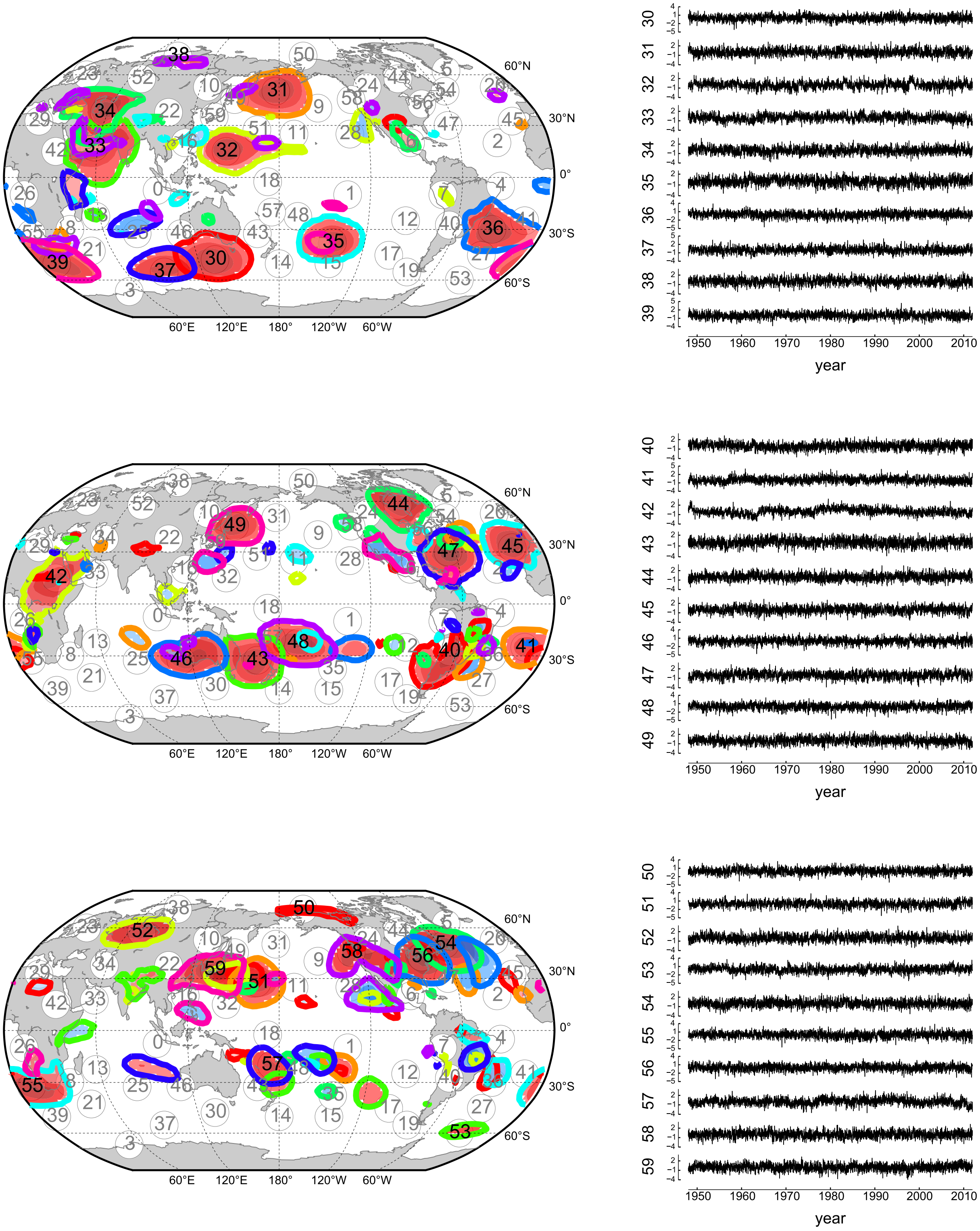

Supplementary Figure 2: Loadings and standardized time series of all components (continued). Not all components are regionally confined, for example No. 53 with loadings in the South Atlantic as well as in the Himalayas.

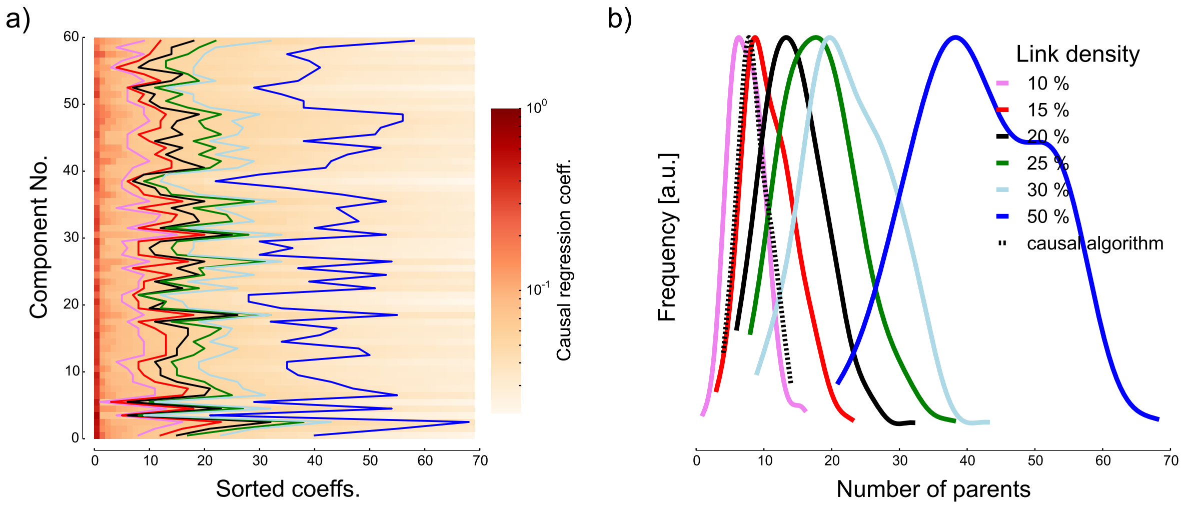

Supplementary Figure 3: Causal regression coefficients and distribution of parents at different network link densities. (a) For each component, the first 70 sorted causal regression coefficients according to Eq. (1) in main article Methods section are shown (absolute value on logarithmic colorbar). The colored lines identify how many of the 'links' are used in the causal effect quantification step for different link densities: 10% (violet), 15% (red), 20% (black), 25% (green), 30% (light blue), 50% (blue). The sharp decay of regression coefficients indicates that typically the first few 'drivers' are by far stronger than the remaining ones. (b) Frequency of number of parents in the time series graph for all considered link densities. The black dashed line shows the density of parents estimated with the causal algorithm which is used only to estimate the causal regression coefficients (see main text).

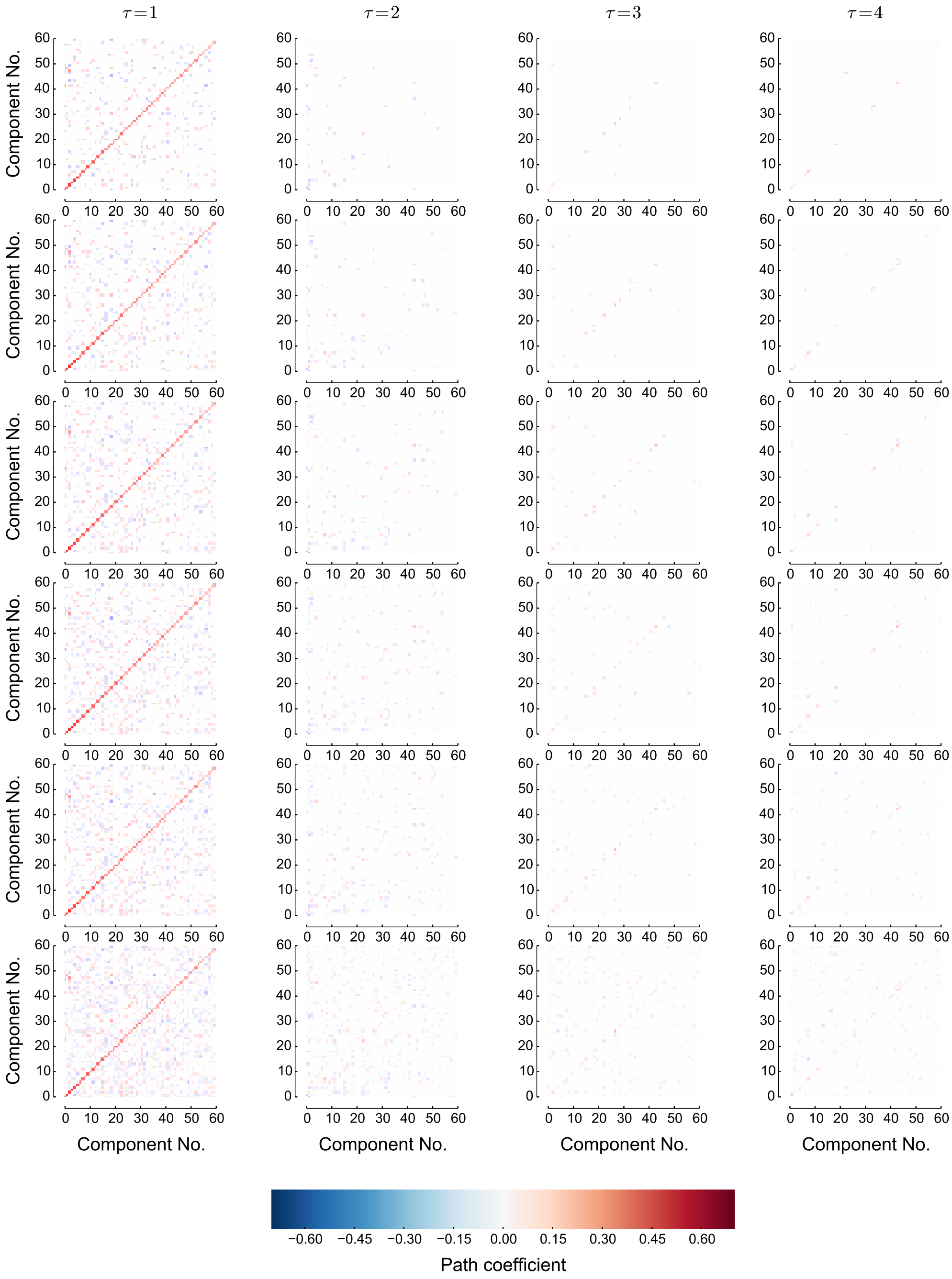

Supplementary Figure 4: Direct causal effects for all component pairs and lags. Shown are the path coefficient matrices with non-zero entries $\mathbf{\Phi}_{ji}(\tau)$ (row j, column i) for every link $X^i_{t-\tau} \to X^j_t$ in the time series graphs at different link densities (10%, 15%, 20%, 25%, 30%, 50%, from top to bottom). Note that the link density refers to the aggregated causal network over all lags where self-links are not counted and at most one link is counted per pair if multiple lags are present. Path coefficients quantify *direct* causal effects. The number of direct links strongly declines for larger lags, here we only consider lags up to $\tau_{\max} = 4$ weeks.

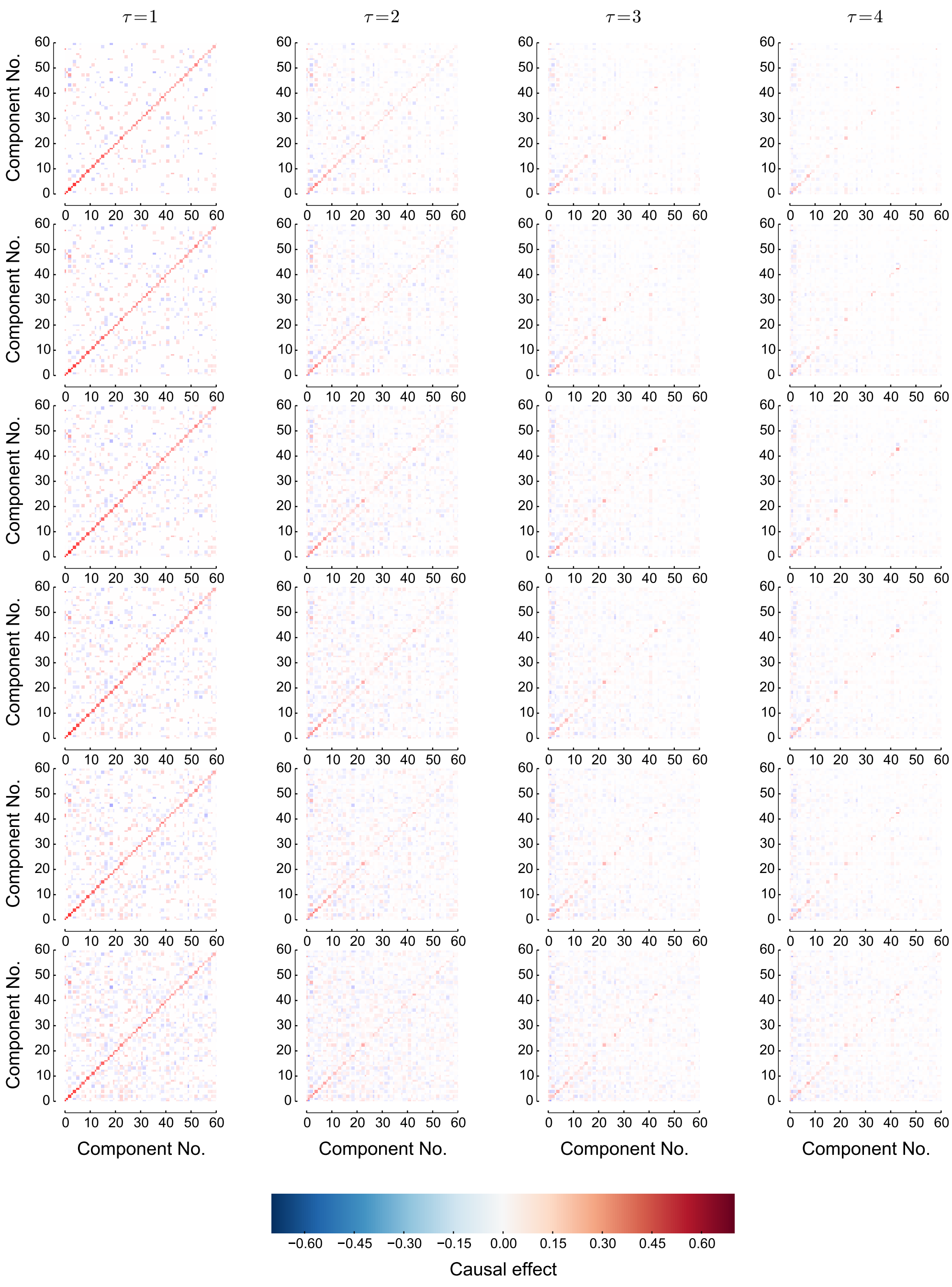

Supplementary Figure 5: Direct and indirect causal effects for all component pairs and lags. Shown are causal effect matrices $\mathbf{\Psi}(\tau)$ quantifying also *indirect* effects, computed from the path coefficient matrices according to main article Eq. (6) at different link densities (10%, 15%, 20%, 25%, 30%, 50%, from top to bottom). An entry $\mathbf{\Psi}_{ji}(\tau)$ refers to the causal effect $I^{\mathrm{CE}}_{i\to j}(\tau)$. Note that $\mathbf{\Psi}(1) = \mathbf{\Phi}(1)$. The strength strongly declines for larger lags.

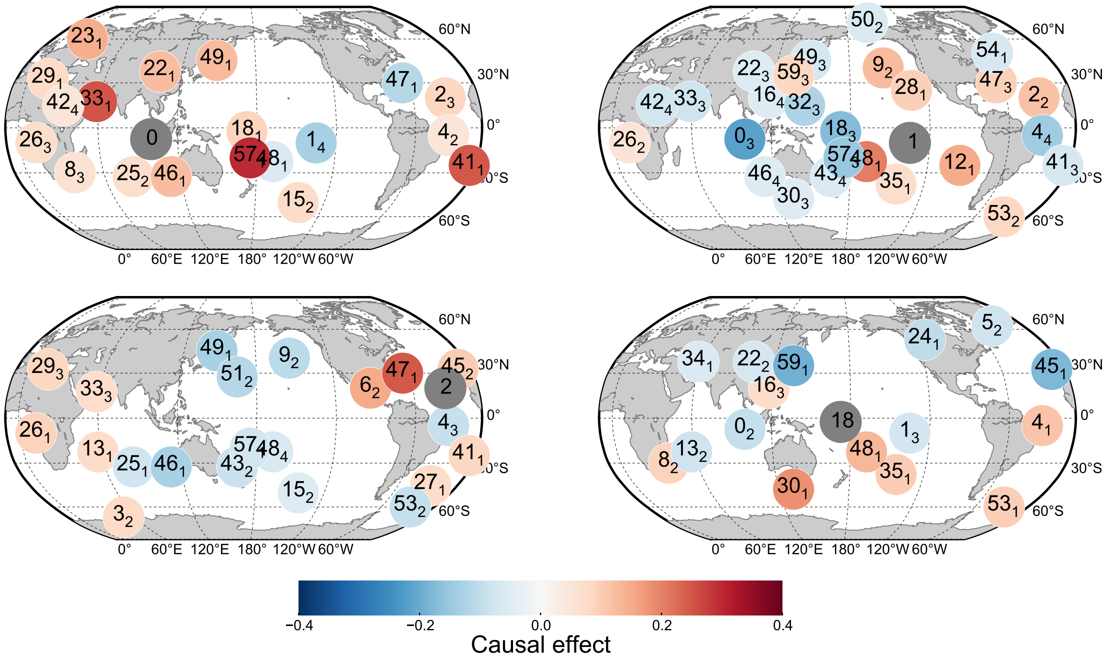

Supplementary Figure 6: Causal effects emanating from components No. 0, 1, 2, 18 (gray nodes in the four panels). The colors denote the causal effect $I^{\mathrm{CE}}_{i\to j}(\tau)$ at the lag with maximum absolute effect (shown as subscript). Only affected nodes with $|I^{\mathrm{CE,\,max}}| > 0.05$ are drawn. Interestingly, while the Indo-Pacific components No. 0, 1, and 18 are strongly interlinked, the Atlantic component No. 2 does not affect the other major equatorial components. Some influences can be explained by the global Walker circulation (see Fig. 4(e) in the main article), for example, the effect of No. 0 to the west in the Arabian Sea (No. 33) and to the East in the equatorial Pacific (No. 18, 1, 57) [1, 2]. An interesting observation is the causal effect of No. 18 on the North Atlantic Oscillation (No. 5), which could be related to the teleconnection of the West Pacific towards the North Atlantic that has recently gained a lot of interest [3].

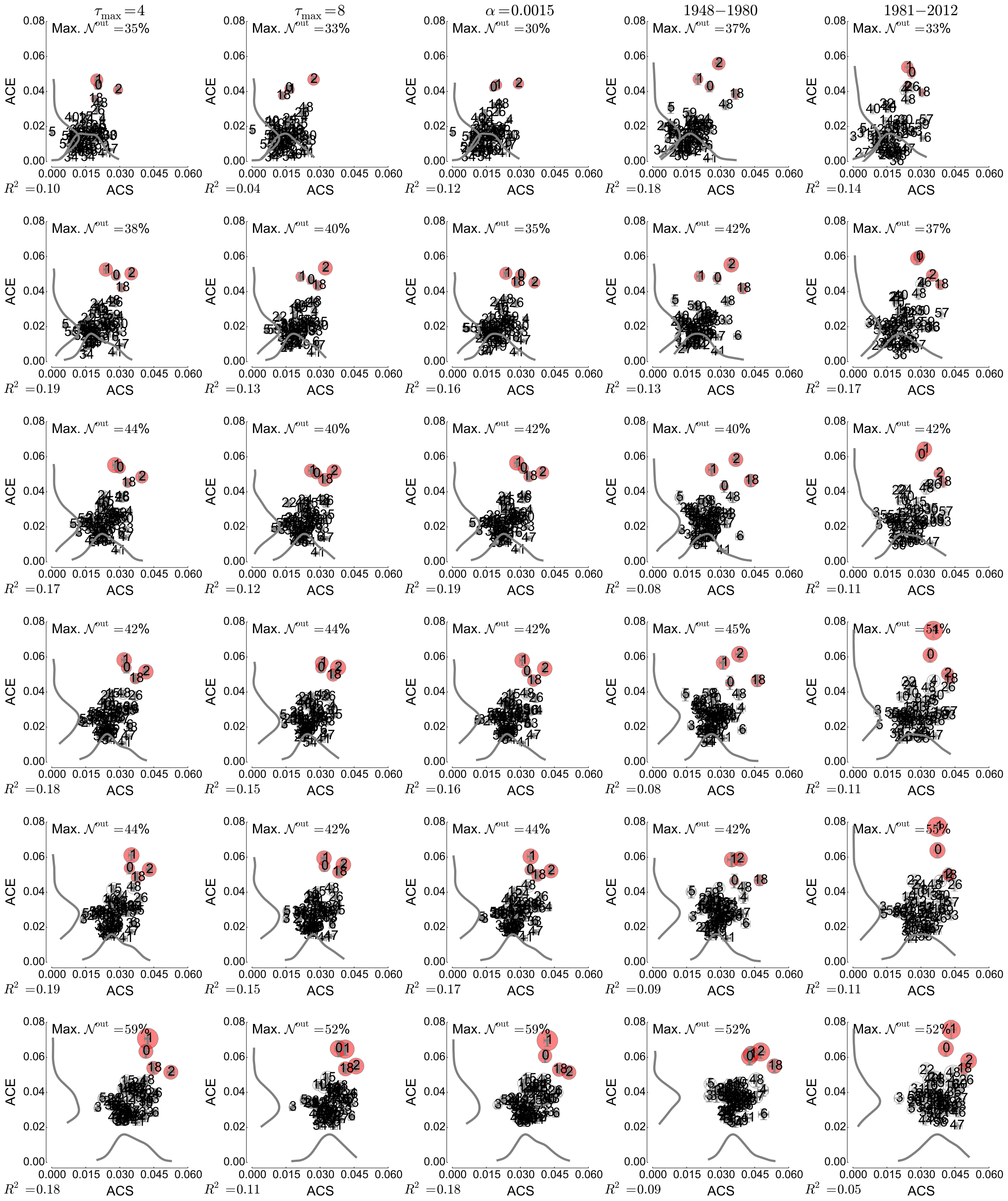

Supplementary Figure 7: Robustness of results for aggregated measures ACE and ACS. Each panel corresponds to main article Fig. 4(c) for different link densities (10%, 15%, 20%, 25%, 30%, 50%, from top to bottom) and other reconstruction parameters, from left to right: maximum time lag $\tau_{\max}$, significance level α in the causal algorithm, and stationary of results for first (1948–1980) and second half (1981–2012) of dataset. In the main article, covering the whole dataset 1948–2012, the parameters are $\tau_{\max} = 4$ weeks and $\alpha = 0.001$. Clearly, $\mathcal{N}^{\text{out}}$ (node size) and ACE / ACS values become larger for denser networks since more paths through weak links are taken into account, but, importantly, the overall rank of the stronger components (red markers) regarding ACE and, to a lesser degree also ACS, is very robust.

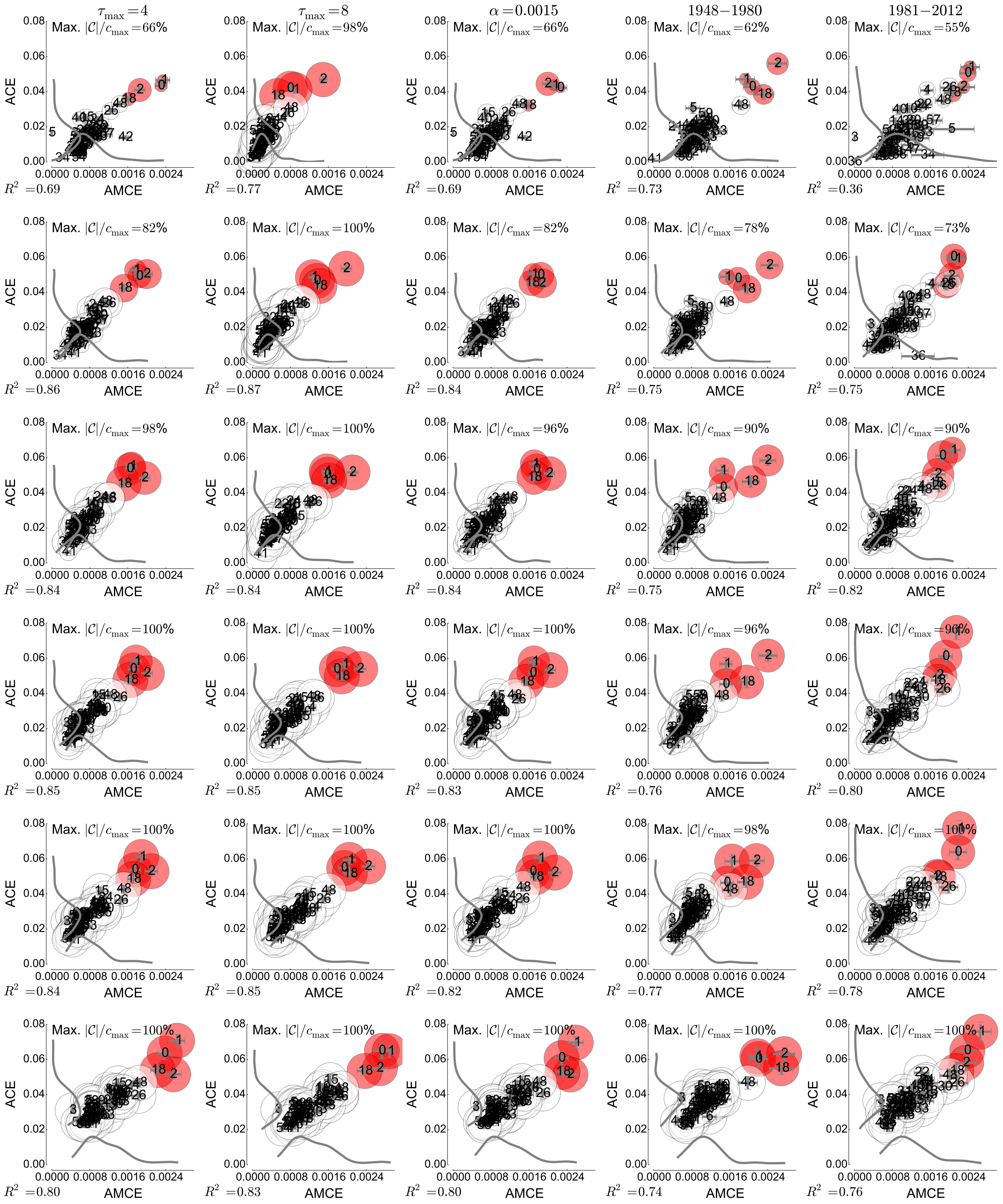

Supplementary Figure 8: Robustness of results for aggregated measures ACE and AMCE. As in Supplementary Fig. 7, but for the main article Fig. 4(d). Changing the time lag or significance level has little effect on ACE values which are comparable within error bounds. Larger differences are observed between the two periods 1948–1980 and 1981–2012. For example, in the second half, the East Pacific ENSO component No. 1 becomes much stronger. Also AMCE is relatively robust regarding the rank of the strong components No. 0, 1, 2, and 18. Interestingly, in the second half 1981–2012 component No. 48 in the tropical Southwest Pacific becomes a more dominant mediator. For denser networks almost all components are a mediator between any other pair ($|\mathcal{C}_k|/c_{\max} \approx 100\,\%$), but most components are only weakly contributing.

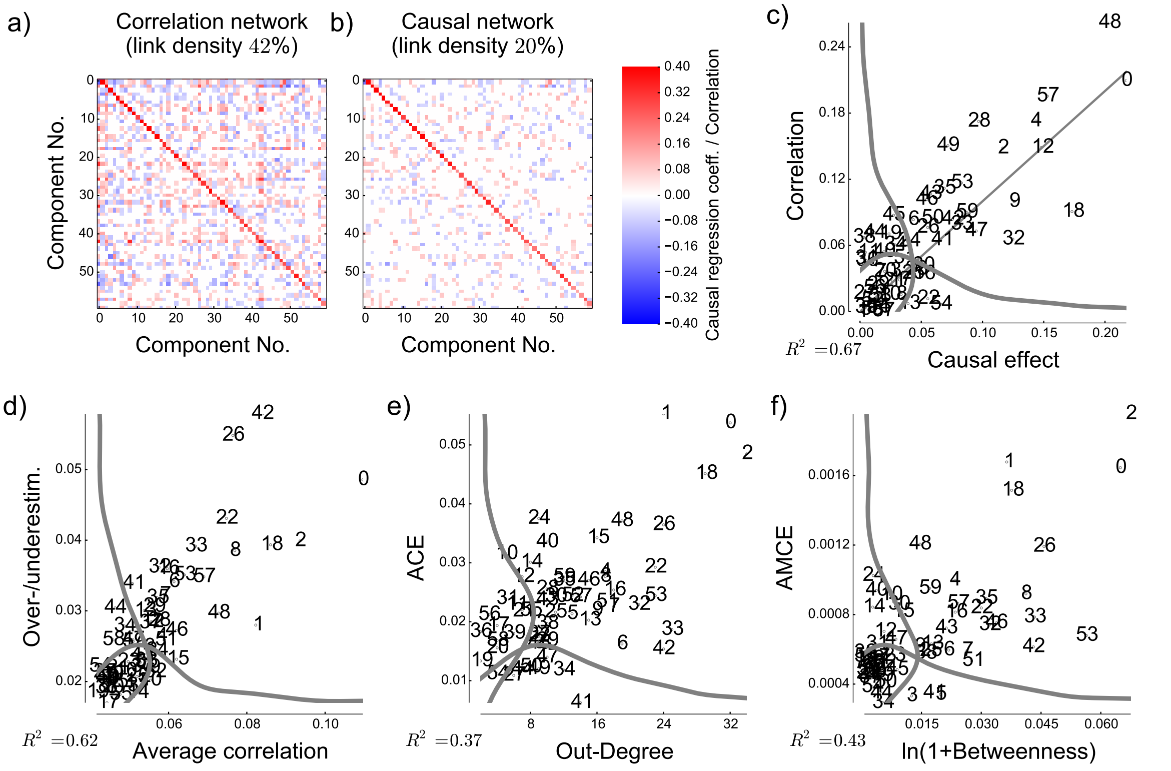

(a)

Supplementary Figure 9: Comparison of results with correlation analysis and classical network measures. (a) Pairwise non-causal correlation matrix with entries $r_{ij}(\tau) = r(X^i_{t-\tau}, X^j_t)$ and (b) causal network defined by thresholded causal regression matrix $C_{i\to j}(\tau)$ (main article Eq. (1)), both collapsed to value at lag of absolute maximum (also for auto-links on the diagonal). For visual comparison, here the non-causal correlation matrix is thresholded with the same threshold as the causal network ($\theta = 0.0585$) resulting in a link density of 42% (not counting self links). (c) Causal effect $I^{\mathrm{CE,\,max}}_{1\to j}$ emanating from component No. 1 representing the East Pacific central ENSO region plotted against the correlation $|r_{1j}|$ at the same respective lag where $I^{\mathrm{CE}}_{1\to j}(\tau)$ takes its absolute maximum. (d) gives the average of a correlation $\frac{1}{N-1}\sum_{j\neq i}|r_{ij}|$ taken at the same lag as the corresponding $I^{\mathrm{CE,\,max}}$ versus $\frac{1}{N-1}\sum_{j\neq i}|I^{\mathrm{CE,\,max}}_{i\to j} - r_{ij}|$ to highlight how much a non-causal correlation over- or underestimates the causal effect of perturbations. For the analysis of network measures, we threshold the correlation matrix to the same link density (20%) as the causal network for better comparability. Note that this non-causal network is still directed. (e) shows ACE versus the node's (out-)degree and (f) AMCE versus the node's shortest-path betweenness centrality (logged with base e) in the non-causal network. In all scatter plots, the gray curves denote the density of the marginal distributions (arbitrary units) and R^2 in the lower left corner denotes the explained variance between both quantities.

Supplementary Table 1: Robustness results for the teleconnection between ENSO and the Indian Ocean. The results for different causal network link densities and other reconstruction parameters are shown: significance level α in the causal algorithm, and stationary of results for first (1948–1980) and second half (1981–2012) of the dataset. The number in brackets denotes the error of the last digit. In the main article, covering the whole dataset 1948–2012, we use $\alpha = 0.001$. While the mediated causal effect of about -0.05 is extremely robust for different link densities or the significance level, the total causal effect varies slightly more, but is largely robust for denser networks at around -0.08. Regarding stationarity, however, the mediation mechanism is much stronger (≈ -0.08) in the second half of the dataset 1981–2012 than in the first half (≈ -0.03).

	link density					
	10%	15%	20%	25%	30%	50%
Main analysis						
CE	−0.052(7)	−0.052(7)	−0.08(1)	−0.08(1)	−0.09(1)	−0.09(2)
MCE	−0.046(6)	−0.044(5)	−0.053(6)	−0.053(6)	−0.053(6)	−0.061(8)
in % of CE	89%	84%	64%	62%	60%	68%
$\alpha = 0.0005$						
CE	−0.051(6)	−0.045(7)	−0.08(1)	−0.08(1)	−0.09(1)	−0.09(2)
MCE	−0.044(5)	−0.046(5)	−0.047(6)	−0.052(6)	−0.055(6)	−0.061(9)
in % of CE	87%	101%	62%	62%	61%	66%
$\alpha = 0.0015$						
CE	−0.048(5)	−0.050(7)	−0.08(1)	−0.09(1)	−0.09(2)	−0.09(2)
MCE	−0.047(5)	−0.043(5)	−0.056(7)	−0.058(6)	−0.057(6)	−0.062(8)
in % of CE	97%	84%	65%	65%	61%	69%
1948 − 1980						
CE	−0.08(2)	−0.06(3)	−0.05(2)	−0.05(3)	−0.04(3)	−0.03(3)
MCE	−0.027(6)	−0.028(6)	−0.027(6)	−0.026(6)	−0.030(6)	−0.029(7)
in % of CE	32%	43%	54%	53%	76%	86%
1981 − 2012						
CE	−0.09(1)	−0.10(2)	−0.09(2)	−0.09(2)	−0.09(2)	−0.05(2)
MCE	−0.08(1)	−0.061(9)	−0.07(1)	−0.08(1)	−0.09(1)	−0.06(1)
in % of CE	85%	61%	76%	87%	95%	113%

Supplementary Table 2: Parents $\mathcal{P}$ for all components estimated with causal algorithm. Parents are ordered by their partial correlation with the respective component conditional on the remaining parents. These parents are used in the causal regression (Eq. (1) in the main article), but are not to be confused with the parents in the time series graph which is based on thresholding the causal regression matrix (Eq. (2) in the main article, Supplementary Fig. 3(a)). The parents in the time series graph can be read off from Supplementary Fig. 4 at different link densities.

$\mathcal{P}_0 = (0_{t-1}, 1_{t-2}, 40_{t-1}, 22_{t-1}, 0_{t-3}, 18_{t-1}, 56_{t-1}, 21_{t-1}, 42_{t-2}, 8_{t-2}, 5_{t-3})$
$\mathcal{P}_1 = (1_{t-1}, 0_{t-2}, 4_{t-1}, 22_{t-1}, 1_{t-4}, 38_{t-1}, 52_{t-1}, 59_{t-1}, 53_{t-1}, 55_{t-1}, 40_{t-1}, 13_{t-1})$
$\mathcal{P}_2 = (2_{t-1}, 26_{t-1}, 57_{t-1}, 22_{t-1}, 15_{t-1}, 28_{t-1}, 51_{t-1}, 1_{t-2}, 12_{t-1}, 8_{t-1}, 55_{t-1}, 29_{t-1})$
$\mathcal{P}_3 = (3_{t-1}, 37_{t-1}, 24_{t-1}, 4_{t-4}, 15_{t-2}, 34_{t-1})$
$\mathcal{P}_4 = (4_{t-1}, 18_{t-1}, 1_{t-2}, 38_{t-1}, 2_{t-2}, 15_{t-1}, 48_{t-1}, 57_{t-1}, 8_{t-2}, 4_{t-2}, 31_{t-2})$
$\mathcal{P}_5 = (5_{t-1}, 29_{t-1}, 18_{t-2}, 23_{t-2})$
$\mathcal{P}_6 = (6_{t-1}, 48_{t-1}, 11_{t-1}, 24_{t-1}, 59_{t-2}, 6_{t-4}, 9_{t-2}, 55_{t-1}, 52_{t-1}, 28_{t-1}, 2_{t-2}, 31_{t-1}, 7_{t-3})$
$\mathcal{P}_7 = (7_{t-1}, 7_{t-4}, 58_{t-1}, 45_{t-1}, 19_{t-1}, 3_{t-1}, 14_{t-1}, 7_{t-2}, 40_{t-1}, 10_{t-2})$
$\mathcal{P}_8 = (8_{t-1}, 42_{t-1}, 19_{t-1}, 8_{t-4}, 18_{t-2}, 57_{t-1}, 13_{t-2}, 49_{t-1}, 43_{t-1}, 56_{t-1}, 54_{t-1})$
$\mathcal{P}_9 = (9_{t-1}, 52_{t-1}, 5_{t-1}, 28_{t-1}, 2_{t-1}, 1_{t-2}, 12_{t-1}, 24_{t-1})$
$\mathcal{P}_{10} = (10_{t-1}, 23_{t-1}, 59_{t-1}, 52_{t-2}, 16_{t-1}, 29_{t-1}, 5_{t-1}, 37_{t-1}, 11_{t-1})$
$\mathcal{P}_{11} = (11_{t-1}, 7_{t-1}, 31_{t-1}, 6_{t-2}, 20_{t-1}, 11_{t-4}, 15_{t-1}, 28_{t-3})$
$\mathcal{P}_{12} = (12_{t-1}, 1_{t-1}, 15_{t-1}, 35_{t-1}, 37_{t-1}, 22_{t-2}, 25_{t-1})$
$\mathcal{P}_{13} = (13_{t-1}, 27_{t-1}, 40_{t-1}, 31_{t-1}, 37_{t-1}, 26_{t-1}, 18_{t-2}, 29_{t-1}, 23_{t-1}, 25_{t-1}, 22_{t-1})$
$\mathcal{P}_{14} = (14_{t-1}, 58_{t-1}, 3_{t-1}, 32_{t-2}, 11_{t-1}, 7_{t-1}, 22_{t-1}, 25_{t-1}, 39_{t-2}, 9_{t-1})$
$\mathcal{P}_{15} = (15_{t-1}, 43_{t-1}, 21_{t-1}, 30_{t-1}, 2_{t-1}, 15_{t-3}, 46_{t-1}, 28_{t-1}, 14_{t-1}, 7_{t-4})$
$\mathcal{P}_{16} = (16_{t-1}, 53_{t-1}, 10_{t-1}, 59_{t-1}, 6_{t-1}, 20_{t-1}, 33_{t-1}, 32_{t-1}, 12_{t-1}, 18_{t-3}, 34_{t-3})$
$\mathcal{P}_{17} = (17_{t-1}, 30_{t-1}, 32_{t-1}, 35_{t-1}, 21_{t-1}, 19_{t-1}, 47_{t-1}, 51_{t-1}, 3_{t-1})$
$\mathcal{P}_{18} = (18_{t-1}, 1_{t-2}, 18_{t-4}, 48_{t-1}, 0_{t-1}, 28_{t-3}, 51_{t-1}, 7_{t-3}, 12_{t-1}, 30_{t-1}, 38_{t-1}, 55_{t-1}, 52_{t-1})$
$\mathcal{P}_{19} = (19_{t-1}, 40_{t-1}, 30_{t-1}, 14_{t-1}, 12_{t-1}, 23_{t-1}, 38_{t-1})$
$\mathcal{P}_{20} = (20_{t-1}, 16_{t-1}, 7_{t-1}, 56_{t-1}, 5_{t-1}, 13_{t-1}, 19_{t-1}, 37_{t-1})$
$\mathcal{P}_{21} = (21_{t-1}, 48_{t-1}, 15_{t-1}, 31_{t-1}, 46_{t-1}, 16_{t-1})$
$\mathcal{P}_{22} = (22_{t-1}, 10_{t-1}, 11_{t-2}, 40_{t-1}, 8_{t-2}, 22_{t-2}, 22_{t-3}, 34_{t-1}, 37_{t-1}, 12_{t-1})$
$\mathcal{P}_{23} = (23_{t-1}, 0_{t-1}, 48_{t-1}, 20_{t-1}, 47_{t-1}, 52_{t-1}, 43_{t-1}, 58_{t-2})$
$\mathcal{P}_{24} = (24_{t-1}, 9_{t-1}, 44_{t-1}, 53_{t-1}, 51_{t-1}, 31_{t-1}, 18_{t-1}, 40_{t-2})$
$\mathcal{P}_{25} = (25_{t-1}, 33_{t-1}, 13_{t-1}, 2_{t-1}, 55_{t-1}, 35_{t-1}, 19_{t-2}, 14_{t-1})$
$\mathcal{P}_{26} = (26_{t-1}, 15_{t-1}, 22_{t-1}, 26_{t-3}, 8_{t-1}, 40_{t-1}, 57_{t-1}, 30_{t-1}, 47_{t-2}, 2_{t-1}, 45_{t-1}, 16_{t-2}, 27_{t-2})$
$\mathcal{P}_{27} = (27_{t-1}, 14_{t-1}, 2_{t-1}, 59_{t-1}, 33_{t-1})$
$\mathcal{P}_{28} = (28_{t-1}, 28_{t-3}, 1_{t-1}, 58_{t-1}, 47_{t-1}, 37_{t-1}, 59_{t-1}, 54_{t-1}, 57_{t-3}, 30_{t-1})$
$\mathcal{P}_{29} = (29_{t-1}, 8_{t-1}, 21_{t-1}, 43_{t-1}, 0_{t-1}, 27_{t-1}, 56_{t-1}, 2_{t-3})$
$\mathcal{P}_{30} = (30_{t-1}, 14_{t-1}, 38_{t-1}, 33_{t-1}, 18_{t-1}, 46_{t-1}, 53_{t-1}, 52_{t-1})$

Supplementary Table 3: Parents $\mathcal{P}$ for all components (continued).

$\mathcal{P}_{31} = (31_{t-1}, 58_{t-1}, 5_{t-1}, 43_{t-1}, 7_{t-1}, 40_{t-2})$
$\mathcal{P}_{32} = (32_{t-1}, 40_{t-1}, 13_{t-1}, 21_{t-1}, 32_{t-3}, 24_{t-1}, 12_{t-1}, 1_{t-2}, 32_{t-4}, 9_{t-1}, 48_{t-1})$
$\mathcal{P}_{33} = (33_{t-1}, 50_{t-1}, 0_{t-1}, 33_{t-4}, 8_{t-2}, 31_{t-2}, 53_{t-1}, 2_{t-2}, 59_{t-1}, 3_{t-1}, 9_{t-1}, 55_{t-2})$
$\mathcal{P}_{34} = (34_{t-1}, 16_{t-1}, 48_{t-1}, 50_{t-1}, 46_{t-1})$
$\mathcal{P}_{35} = (35_{t-1}, 33_{t-1}, 36_{t-1}, 15_{t-1}, 24_{t-1}, 1_{t-1}, 48_{t-1}, 51_{t-1}, 49_{t-2}, 14_{t-1}, 38_{t-1})$
$\mathcal{P}_{36} = (36_{t-1}, 25_{t-1}, 14_{t-1}, 32_{t-1}, 4_{t-1}, 42_{t-2}, 45_{t-2})$
$\mathcal{P}_{37} = (37_{t-1}, 39_{t-1}, 28_{t-1}, 43_{t-1}, 26_{t-1}, 17_{t-1}, 46_{t-1}, 37_{t-3})$
$\mathcal{P}_{38} = (38_{t-1}, 10_{t-1}, 51_{t-1}, 46_{t-1}, 5_{t-1}, 20_{t-1}, 56_{t-1})$
$\mathcal{P}_{39} = (39_{t-1}, 37_{t-1}, 27_{t-1}, 54_{t-1}, 33_{t-1}, 12_{t-1}, 36_{t-1}, 39_{t-4}, 51_{t-1})$
$\mathcal{P}_{40} = (40_{t-1}, 17_{t-1}, 24_{t-1}, 36_{t-1}, 2_{t-3}, 20_{t-2}, 32_{t-1}, 40_{t-4}, 40_{t-3})$
$\mathcal{P}_{41} = (41_{t-1}, 55_{t-1}, 0_{t-1}, 5_{t-1}, 35_{t-1}, 3_{t-1}, 2_{t-1}, 52_{t-1})$
$\mathcal{P}_{42} = (42_{t-1}, 57_{t-1}, 15_{t-1}, 42_{t-4}, 42_{t-2}, 42_{t-3}, 29_{t-1}, 8_{t-1}, 43_{t-1}, 45_{t-1}, 47_{t-3}, 55_{t-1}, 56_{t-1}, 23_{t-3})$
$\mathcal{P}_{43} = (43_{t-1}, 46_{t-1}, 24_{t-1}, 2_{t-1}, 14_{t-1}, 40_{t-1}, 43_{t-4}, 36_{t-1}, 30_{t-1})$
$\mathcal{P}_{44} = (44_{t-1}, 31_{t-1}, 12_{t-1}, 22_{t-1}, 15_{t-1}, 29_{t-1}, 25_{t-1})$
$\mathcal{P}_{45} = (45_{t-1}, 18_{t-1}, 49_{t-1}, 4_{t-2}, 2_{t-1})$
$\mathcal{P}_{46} = (46_{t-1}, 2_{t-1}, 21_{t-1}, 37_{t-1}, 0_{t-1}, 14_{t-1}, 25_{t-1}, 16_{t-1})$
$\mathcal{P}_{47} = (47_{t-1}, 2_{t-1}, 0_{t-1}, 7_{t-1}, 9_{t-1}, 20_{t-1})$
$\mathcal{P}_{48} = (48_{t-1}, 35_{t-1}, 1_{t-1}, 39_{t-1}, 13_{t-1}, 18_{t-1}, 26_{t-2}, 25_{t-1})$
$\mathcal{P}_{49} = (49_{t-1}, 2_{t-1}, 0_{t-1}, 48_{t-1}, 53_{t-1}, 24_{t-1})$
$\mathcal{P}_{50} = (50_{t-1}, 10_{t-1}, 5_{t-1}, 47_{t-1}, 44_{t-1}, 1_{t-2}, 35_{t-2})$
$\mathcal{P}_{51} = (51_{t-1}, 26_{t-1}, 11_{t-1}, 2_{t-2}, 56_{t-1})$
$\mathcal{P}_{52} = (52_{t-1}, 4_{t-1}, 59_{t-1}, 55_{t-1}, 45_{t-1}, 38_{t-1}, 56_{t-1})$
$\mathcal{P}_{53} = (53_{t-1}, 17_{t-1}, 18_{t-1}, 2_{t-2}, 12_{t-1}, 35_{t-1}, 3_{t-2}, 53_{t-4}, 8_{t-3})$
$\mathcal{P}_{54} = (54_{t-1}, 24_{t-1}, 6_{t-1}, 56_{t-1}, 49_{t-1}, 42_{t-1}, 50_{t-1}, 7_{t-1}, 9_{t-1})$
$\mathcal{P}_{55} = (55_{t-1}, 5_{t-1}, 46_{t-1}, 53_{t-1})$
$\mathcal{P}_{56} = (56_{t-1}, 23_{t-1}, 35_{t-1}, 50_{t-1}, 45_{t-1}, 13_{t-1}, 14_{t-1})$
$\mathcal{P}_{57} = (57_{t-1}, 0_{t-1}, 48_{t-1}, 2_{t-1}, 6_{t-1}, 57_{t-4}, 1_{t-2})$
$\mathcal{P}_{58} = (58_{t-1}, 38_{t-1}, 15_{t-1}, 31_{t-1}, 57_{t-1}, 33_{t-1}, 17_{t-1})$
$\mathcal{P}_{59} = (18_{t-1}, 59_{t-1}, 30_{t-1}, 31_{t-1}, 4_{t-1}, 19_{t-2}, 26_{t-1}, 16_{t-3})$

Supplementary Note 1 Comparison with correlation analysis and classical network measures

Complex network analysis is a very active field of current research and has been applied to study the structure and function of complex systems in neuroscience (functional networks) [4–7] and more recently also in climate research (climate networks) [8–14]. As opposed to transportation systems or financial markets where at least the network's nodes and often also the links are given (for example, by physical traffic connections), in the brain (especially on a macroscale) and the Earth system the network's nodes and links first need to be suitably defined. While the grid locations of measurements suggest themselves as nodes, these are often not the variables of interest. Regarding the links, especially in neuroscience different definitions have emerged from structural connectivity describing white matter tracts within the brain [15] to functional connectivity [4], where the links are obtained from similarities between time series from grid point locations (for example, voxels in functional magnetic resonance imaging measurements). Similarities are typically estimated using pairwise association measures such as Pearson correlation or mutual information leading to an association matrix which is thresholded. The resulting binary adjacency matrix is studied from a graph-theoretical perspective using statistical network theory [16]. Local node properties such as the node degree are used to determine *hubs* in the network and more global properties such as *betweenness centrality* [17] based on shortest path lengths are typically associated with a node's importance for 'information transfer', for example, in the brain [5], but also in the climate system [10].

However, from a theoretical standpoint classical network reconstruction methods do not allow for a causally connotated interpretation of perturbation or information transfer. A first problem is that the frequently applied zero-lag association measures do not allow for any directional interpretation of an association. A directionality can be based on Granger causality [18] which has been used to study Granger-causal interactions among small sets of climatic variables [19–23], but also to reconstruct directional networks using an information-theoretic generalization [23]. However, if these approaches are based on bivariate pairwise analyses, they can to some extent address the question of directionality between two nodes, but not the problem of causal interactions among multiple nodes (see main article Fig. 1). Spurious links due to common drivers and transitivity effects (main article Fig. 1(**c**)) present a strong bias and the authors in Refs. [24, 25] have shown that even for a set of entirely independent processes a small-world topology (small average path length and high clustering of the network) of the network emerges.

In the following, we compare the results for the causal effect measures to a much simpler non-causal correlation-based analysis. In Supplementary Fig. 9(a) we depict this correlation network with the same significance threshold applied as for the causal network at 20% link density, which results in a more than twice as large link density. In Supplementary Fig. 9(c), we compare the correlation of component No. 1 with all other components at the same lag as the maximum causal effect $I_{1\to j}^{\mathrm{CE,\,max}}$. The explained variance between the two is $R^2 = 0.67$, but there are substantial differences. Mainly, the correlations overestimate the causal effect, for some components like No. 28 in East Africa the corresponding correlation is twice as large as the causal effect. A larger correlation indicates that common driver nodes exert an effect with the same sign as the causal effect while for weaker correlation values the effect is anti-correlated. In Supplementary Fig. 9(d) we analyze the average difference over all components by plotting how much a non-causal correlation over- or underestimates the causal effect against the average correlation value. Among the major global gateway components No. 0, 1, 2, 18, the effect of No. 0 is most strongly overestimated by about 50% of the average correlation value. No. 42 here ranks among the largest components according to its average correlation value even though it has only a very low ACE value (Supplementary Fig. 9(e)).

For similar reasons also the classical network measures previously used in, for example, Refs. [8, 10, 26–28], give different results. The non-causal network with adjacency matrix A of shape (N, N) is obtained from the lagged correlation matrix $r_{ij}(\tau) = r(X^i_{t-\tau}, X^j_t)$, collapsed to only the lag $(\tau > 0)$ with largest absolute correlation, $A_{ij} = 1$ if $\max_{\tau>0} |r_{ij}(\tau)| > \theta^*$ and $A_{ij} = 0$ else, where θ^* is the threshold

yielding the same link density as in the causal network (20%). Note that this correlation-based network is still directed. In Supplementary Fig. 9(e) we highlight that a network measure like the (out-)degree of the non-causal network allows to roughly classify components with weak or strong causal effect, but is not very predictive on a more detailed level (explains only about $R^2 \approx 40\%$). For example, No. 1 and 33 have the same degree, while the latter's ACE is much larger. These results underline that simple correlation-based network analyses carry not much predictive information for the actual effect of perturbations and show the need for sound causal measures to confidently estimate the possible influence of perturbations.

Finally, Supplementary Fig. 9(f) underlines the importance of causal quantitative measures of mediative effect as opposed to classical network measures based on the correlation matrix to measure the transfer of perturbations: The betweenness centrality of a component in the corresponding non-causal network (with equal link density 20%) is not very predictive ($R^2 \approx 40\%$) for mediating causal effect (except for a rough classification into weak or strong AMCE), even components with only average AMCE like the Arabian Sea component No. 33 or 42 in West Africa have the same betweenness value as No. 1. Another variant for an analogous measure to betweenness centrality can be obtained by normalizing each summand in the main article Eq. (13) by the corresponding causal effect,

$$I_k^{\mathrm{AMCE,\,norm}} = \frac{1}{|\mathcal{C}_k|} \sum_{(i,j)\in\mathcal{C}_k} \max_{\tau} \frac{|I^{\mathrm{MCE}}_{i\to j|k}(\tau)|}{|I^{\mathrm{CE}}_{i\to j}(\tau)|}\,,$$

which is, however, not robust to outliers [29], especially for small CE.

Supplementary Note 2 Causal algorithm

Here the causal algorithm is only used as a variable selection to obtain the parents for the causal regression estimation in main article Eq. (1). The main idea is to iteratively unveil the parents of a subprocess $Y \in \mathbf{X}$ by testing for conditional independence between Y_t and each remaining component at a range of time lags conditioned on an iteratively increasing set of the other remaining components: For every Y, first we estimate the correlations $\rho(X_{t-\tau}; Y_t)$ and initialize the preliminary parents $\mathcal{P}_{Y_t} = \{X_{t-\tau} : X \in \mathbf{X}, 0 < \tau \leq \tau_{\max}, \rho(X_{t-\tau}; Y_t) \neq 0\}$. This set contains also indirect links which are now iteratively removed by testing whether the dependence between Y_t and each $X_{t-\tau} \in \mathcal{P}_{Y_t}$, conditioned on the incrementally increased set of conditions $\mathcal{P}^{n,i}_{Y_t} \subseteq \mathcal{P}_{Y_t}$ of cardinality n, vanishes:

> Iterate n over increasing number of conditions, starting with some $n_0 \geq 1$:
>
> > Iterate i through all combinations of picking n conditions from $\mathcal{P}_{Y_t}$ to define $\mathcal{P}^{n,i}_{Y_t}$ in this step.
> >
> > > For all $X_{t-\tau} \in \mathcal{P}_{Y_t}$, estimate the partial correlation $\rho(X_{t-\tau}; Y_t \,|\, \mathcal{P}^{n,i}_{Y_t})$. If $\rho(X_{t-\tau}; Y_t \,|\, \mathcal{P}^{n,i}_{Y_t}) = 0$, remove $X_{t-\tau}$ from $\mathcal{P}_{Y_t}$.
> >
> > The iteration over i stops if all possible combinations have been tested. (As detailed below, in the implementation we check relevant combinations of conditions first and test up to ten combinations.)
>
> If the cardinality $|\mathcal{P}_{Y_t}| \leq n$, the algorithm converges, else, increase n by one and iterate again. (If the initial number of conditions is $n_0 > 1$ to speed up the algorithm, also previously skipped combinations with $n < |\mathcal{P}_{Y_t}|$ need to be checked before convergence can be assessed.)

The main free parameters are the maximum time lag $\tau_{\max}$ and the significance level to determine whether $\rho(X_{t-\tau}; Y_t \,|\, \mathcal{P}^{n,i}_{Y_t}) \neq 0$. To limit computational time [30], we start with an initial number of conditions $n_0 = 3$. To speed up the performance in the i-loop, we first condition on nodes with largest partial correlation in the previous step which are determined after each n-loop by sorting the elements $X_{t-\tau}$ in $\mathcal{P}_{Y_t}$ by the value of $\min_i |\rho(X_{t-\tau}; Y_t \,|\, \mathcal{P}^{n,i}_{Y_t})|$.

Supplementary References

1. Webster, P. J. *et al.* Monsoons: Processes, predictability, and the prospects for prediction. *J. Geophys. Res. Oceans* **103,** 14451–14510 (1998).
2. Hosking, J. S., Russo, M. R., Braesicke, P. & Pyle, J. A. Tropical convective transport and the Walker circulation. *Atmos. Chem. Phys.* **12,** 9791–9797 (2012).
3. Trenberth, K. E., Fasullo, J. T., Branstator, G. & Phillips, A. S. Seasonal aspects of the recent pause in surface warming. *Nat. Clim. Chang.* **4,** 911–916 (2014).
4. Friston, K. J. Functional and effective connectivity in neuroimaging: A synthesis. *Hum. Brain Mapp.* **2,** 56–78 (1994).
5. Bullmore, E. & Sporns, O. Complex brain networks: graph theoretical analysis of structural and functional systems. *Nat. Rev. Neurosci.* **10,** 186–198 (2009).
6. Schinkel, S., Zamora-Lopez, G., Dimingen, O., Sommer, W. & Kurths, J. Functional network analysis reveals differences in the semantic priming task. *J. Neurosci. Methods* **197,** 333–339 (2011).
7. Simpson, S. L., Bowman, F. D. & Laurienti, P. J. Analyzing complex functional brain networks: Fusing statistics and network science to understand the brain. *Statist. Surv.* **7,** 1–36 (2013).
8. Tsonis, A. A., Swanson, K. L. & Wang, G. On the role of atmospheric teleconnections in climate. *J. Climate* **21,** 2990–3001 (2008).
9. Yamasaki, K., Gozolchiani, A. & Havlin, S. Climate networks around the globe are significantly affected by El Niño. *Phys. Rev. Lett.* **100,** 228501 (2008).
10. Donges, J. F., Zou, Y., Marwan, N. & Kurths, J. The backbone of the climate network. *Eur. Phys. J. Spec. Top.* **87,** 48007 (2009).
11. Ebert-Uphoff, I. & Deng, Y. Causal discovery for climate research using graphical models. *J. Climate* **25,** 5648–5665 (2012).
12. Deng, Y. & Ebert-Uphoff, I. Weakening of atmospheric information flow in a warming climate in the Community Climate System Model. *Geophys. Res. Lett.* **41,** 193–200 (2014).
13. Boers, N., Bookhagen, B., Barbosa, H., Marwan, N. & Kurths, J. Prediction of extreme floods in the eastern Central Andes based on a complex networks approach. *Nat. Commun.* **5,** 5199 (2014).
14. Donges, J., Petrova, I., Loew, A., Marwan, N. & Kurths, J. How complex climate networks complement eigen techniques for the statistical analysis of climatological data. *Clim. Dyn.* (2015).
15. Hagmann, P. *et al.* Mapping the structural core of human cerebral cortex. *PLoS Biol.* **6,** 1479–1493 (2008).
16. Newman, M. E. J. *Networks: An Introduction* (Oxford University Press, Oxford, 2010).
17. Freeman, L. C. A Set of Measures of Centrality Based on Betweenness. *Sociometry* **40,** 35–41 (1977).
18. Granger, C. W. J. Investigating causal relations by econometric models and cross-spectral methods. *Econometrica* **37,** 424–438 (1969).
19. Kaufmann, R. K. & Stern, D. I. Evidence for human influence on climate from hemispheric temperature relations. *Nature* **388,** 39–44 (1997).
20. Mosedale, T. J., Stephenson, D. B., Collins, M. & Mills, T. C. Granger causality of coupled climate processes: Ocean feedback on the North Atlantic Oscillation. *J. Climate* **19,** 1182–1194 (2006).
21. Mokhov, I. I. *et al.* Alternating mutual influence of El-Nio/Southern Oscillation and Indian monsoon. *Geophys. Res. Lett.* **38,** L00F04 (2011).

22. Attanasio, A., Pasini, A. & Triacca, U. Granger Causality Analyses for Climatic Attribution. *Atmos. Clim. Sci.* **3,** 515–522 (2013).

23. Hlinka, J. *et al.* Reliability of inference of directed climate networks using conditional mutual information. *Entropy* **15,** 2023–2045 (2013).

24. Bialonski, S., Horstmann, M. T. & Lehnertz, K. From brain to earth and climate systems: Small-world interaction networks or not? *Chaos* **20,** 013134 (2010).

25. Hlinka, J., Hartman, D. & Paluš, M. Small-world topology of functional connectivity in randomly connected dynamical systems. *Chaos* **22,** 033107 (2012).

26. Tsonis, A. A. & Roebber, P. J. The architecture of the climate network. *Physica A* **333,** 497–504 (2004).

27. Donges, J. F., Zou, Y., Marwan, N. & Kurths, J. Complex networks in climate dynamics: Comparing linear and nonlinear network construction methods. *Eur. Phys. J. Spec. Top.* **174,** 157–179 (2009).

28. Donges, J. F., Schultz, H. C. H. & Marwan, N. Investigating the topology of interacting networks. *Eur. Phys. J. B* **84,** 635–651 (2011).

29. Preacher, K. J. & Kelley, K. Effect size measures for mediation models: quantitative strategies for communicating indirect effects. *Psychol. Methods* **16,** 93–115 (2011).

30. Runge, J., Donner, R. & Kurths, J. Optimal model-free prediction from multivariate time series. *Physical Review E* **91,** 052909 (2015).
